# Extracellular vesicles enhance oxidative stress through P38/NF‐kB pathway in ketamine‐induced ulcerative cystitis

**DOI:** 10.1111/jcmm.15397

**Published:** 2020-05-22

**Authors:** Xiao jian Xi, Jin jiang Zeng, Yong Lu, Shao hua Chen, Zhi wen Jiang, Peng jie He, Hua Mi

**Affiliations:** ^1^ Department of Urology First Affiliated Hospital of Guangxi Medical University Nanning China; ^2^ Department of Urology Liuzhou Municipal Liutie Central Hospital Liuzhou China

**Keywords:** extracellular vesicles, ketamine‐induced cystitis, oxidative stress

## Abstract

Long‐term abuse of ketamine causes ketamine‐induced cystitis. The functional alterations of bladder epithelial cells in microenvironment during cystitis remain poorly understood. Here, we explored extracellular vesicles (EV) alteration in ketamine‐induced toxicity. To simulate the high‐concentration ketamine environment in vivo*,* we established an in vitro model of high ketamine using human uroepithelial cells (SV‐HUC‐1). Cell viability and proliferation were assessed to evaluate the effects of various concentrations (0, 0.25, 0.5, 1, 2, 4 and 8 mmol/L) of ketamine on SV‐HUC‐1 cells. The cell supernatant cultured at a concentration (0, 1, 2, 4 mmol/L) of ketamine was selected for EV extraction and identified. Subsequently, we assessed different groups (ketamine, ketamine plus EV blocker, EV, EV plus extracellular vesicles blocker) of oxidative stress and expression of inflammation. Last, luciferase reporter assay was performed to study the transcriptional regulation of EV on the NF‐kB and P38 pathway. The results of our study suggested that treatment with 0, 1, 2 or 4 mmol/L ketamine altered the morphology and secretion capacity of extracellular vesicles. As the concentration of ketamine increased, the average particle size of EV decreased, but the crest size, particle concentration and EV protein increased. Moreover, after the addition of EV blocker, EV secreted at different concentrations were blocked outside the cell membrane, and the degree of oxidative stress decreased. Our study provided evidence that ketamine alters the secretion of EV by directly stimulating cells in inflammation microenvironment and EV play significant roles in intercellular signal communication and the formation of KIC.EV

## 1 INTRODUCTION

Ketamine, a derivative of phencyclidine, has been used as an anaesthetic in clinical and analgesic fields. Due to its intense euphoria and hallucinations, ketamine is prone to abuse by young people in entertainment settings. Chronic abuse often leads to bladder contracture and severe lower urinary tract symptoms, clinically called ketamine‐induced cystitis,^1^ which often manifests as mucosal ulcer with bleeding, similar to interstitium cystitis. Several studies have suggested that ketamine and its metabolites interact with bladder epithelium, leading to human uroepithelial cells’ (SV‐HUC‐1) injury, triggering an oxidative stress cascade and, ultimately, cystitis formation.^2^, ^3^ The current treatment options for KIC include surgery and perfusion therapy among others. However, the available treatment strategies are not ideal for all patients.^4^ A better understanding of the mechanism of KIC occurrence and development can, therefore, aid in the identification of new biomarkers, and the development of new KIC treatment strategies.

Being essential mediators of intercellular communication, extracellular vesicles (EV) are vital in the pathogenesis of many diseases.^5^, ^6^ EV (about 30‐800 nm in diameter), such as exosomes and microvesicles, can affect physiological and pathological conditions through the transfer of various cargo molecules, including proteins, nucleic acids and lipids.^7^ As lipid bilayer membrane vesicles, secretion of extracellular vesicles is closely related to cell viability. Numerous studies have reported the value of EV derived from urine, which now appears to be a very promising and completely non‐invasive source of new information that accurately reflects the pathophysiology of the kidneys and other organ systems.^8^ Besides, EV have been found in the urine of patients with interstitial cystitis and can be used as diagnostic biomarkers.^9^, ^10^ The presence of EV in urine reflects potential cellular processes that may be associated with cystitis.^11^

Accumulating evidence indicates that oxidative stress injury plays an essential role in the prognosis of KIC. High levels of intracellular ROS activated the p38 signalling pathway^12^ and stimulate the expression of nuclear factor kappa B (NF‐kB). NF‐kB, a member of a redox‐sensitive transcription factor family, regulates the expression of pro‐inflammatory factors, including TNF‐α, iNOS and COX‐2.^13^, ^14^ According to Juan et al, ketamine‐induced cystitis enhances the translocation of NF‐kB transport and the expression of cyclooxygenase‐2 (COX‐2) through oxidative stress in rats.^15^ Khalyfa A and his team successfully isolated nanovesicles in the serum of patients with obstructive sleep apnoea, and the morphology of the nanovesicles was similar to that of exosomes. The production of such nanovesicles can be triggered by oxidative stress‐related pathways, which also exacerbates cellular senescence.^16^ Besides, a study led by Mizutani et al^17^ also indicated that Akt and CD9 carried by EV derived from urothelial SV‐HUC‐1 cells may be useful markers for differential diagnosis of urinary tract infections and asymptomatic bacteriuria. We, therefore, postulate that EV play an essential role in the mechanism of KIC, although such research has not been previously reported.

Extracellular vesicles extracted from the urine are susceptible to changes in cellular origin, temperature and pH,^18^ rendering experimental results prone to variation. Therefore, we sought to optimize the extraction and identification of EV using SV‐HUC‐1 cells exposed to high concentrations of ketamine. In this study, we found that ketamine cell‐derived EV can transport associated molecules to alter the physiology of target cells in a specific manner. These findings could allow us to explore the mechanisms of signal transduction and targeted regulation of EV, hence provide a new therapeutic strategy for clinical KIC patients.

## 2 MATERIALS AND METHODS

### 2.1 Cell culture and reagents

The immortalized human bladder epithelial cell line SV‐HUC‐1 was purchased from the Chinese Academy of Sciences cell bank. Cells were cultured in DMEM/F12 (Gibco™) medium supplemented with 10% heat‐inactivated foetal bovine serum (FBS) (Gibco™, Thermo Fisher) and 1% antibiotic (Hyclone). We configured the working fluid to the concentration of ketamine (0, 0.25, 0.5, 1, 2, 4, 8 mmol/L) to select the appropriate dose. To extract pure ketamine‐induced EV, we used DMEM/F12 medium supplemented with 12% exosome‐free serum (SBI, EXO‐FBS‐50A‐1), followed by ketamine hydrochloride injection (Fujian Gutian Pharmaceutical Co., Ltd.) for stimulation of SV‐HUC‐1 cells.

### 2.2 Cell exposure

SV‐HUC‐1 cells (4 × 10^3^/well) were grown in 96‐well plates. At 80% confluence, cells were exposed to different concentrations of ketamine working solution (100 µL/well) for 48 hours. The optimal time is 48 hours, the stimulation time is short, and the secretion of EV is insufficient. The long time will cause the cells to produce a large number of apoptotic bodies, which is not conducive to observation. We detected CCK‐8 cell proliferation assay by cell supernatant. The SV‐HUC‐1 cells (4 × 10^4^/well) were also grown in 24‐well plates. Then, we observed the survival of the cells at different concentrations of ketamine, and proteins from cell lysis were collected for the detection of oxidative stress indicators SOD, MDA and GSH.

### 2.3 ELISA

We used the SOD, MDA and GSH ELISA kit (Nanjing Jiancheng Bioengineering Institute, China) to detect the proteins from cell lysis. Quantitative assays were performed using the corresponding ELISA assay kit according to the manufacturer's protocol. The absorbance was measured at 550, 532 and 412 nm using a microplate reader (Thermo Fisher).

### 2.4 CCK‐8 determination

A Cell Counting Kit‐8 (CCK‐8) assay (Dojindo) was used to evaluate the cytotoxicity of ketamine on SV‐HUC‐1 cells. The CCK‐8 solution (10 µL) added to each well, and the plate was incubated for 2.5 hours, at 37°C and an anaerobic condition of 5% CO_2_. The absorbance was determined at 450 nm.

### 2.5 Isolation of EV

SV‐HUC‐1 cells (4 × 10^5^/flask) were cultured in T75 cell culture flasks (Eppendorf). When the cells reached 80% confluence, the culture was discarded and the cells were washed 3 times with cold PBS (4°C). The purpose of washing cells with PBS was to remove as much EV from FBS as possible. The washed cells were then exposed to different concentrations (0, 1, 2 and 4 mmol/L) of exosome‐free ketamine solution (DMEM/F12 medium + 12% exosome‐free serum (SBI, EXO‐FBS‐50A‐1) + ketamine) for 48 hours. Cell culture media (25 mL each) were harvested from four groups, and the larger diameter particles were first filtered using a filter of 0.8 µmol/L (Sartorius Minisart NML). Extraction was started using the exosome extraction kit (QIAGEN, exoEasy Maxi Kit (20)).

1. The cell culture medium was placed in a spin column, and an equal amount of XBP was also added, which mixed together by inverting several times. Then, it was centrifuged at 500 *g* for 1 minute at 4°C using a Beckman Coulter Allegra X‐15R centrifuge (Beckman Coulter), and the liquid was transferred in the spin column to a centrifuge tube. The spin column was taken out, all the liquid was discarded in the centrifuge tube, and the spin column was put back.
2. 8 mL of XBP was added and centrifuged at 5000 *g* for 5 minutes at 4°C. The tube and all the liquid were discarded, the column was retained, and the column was placed into a new tube.
3. 400 µL of XE was added eluate to the spin column, incubated for 1‐2 minutes, and was centrifuged at 500 *g* for 5 minutes at 4°C. The liquid was collected in the centrifuge tube and placed on the spin column again. They were incubated for another 1‐2 minutes and centrifuged at 5000 *g* for 5 minutes at 4°C. Finally, the liquid (20 µL) in the centrifuge tube was transferred to a new container. 80 µL cold PBS was added to each tube (100 µL/tube) for resuspension, and then, the EV were stored at −80°C until further use.^19^

### 2.6 Protein extraction and quantification

About 40 µL of lysis buffer (RIPA: PMSF = 100:1, Solarbio) was added to each EV sample (40 µL/tube). The EV were lysed on ice for 30 minutes. Protein concentration was then estimated using a BCA kit (Sigma).

### 2.7 Electron microscope

A 20 µL drop of resuspended EV was placed on a sheet of Parafilm. Grids were transferred to the drops of EV for 3 minutes, and then dried from the edge using filter paper (Solarbio). The sample side of the membrane was then transferred to a 30 µL drop of 3% phosphotungstic acid solution, which was negative stained at room temperature for 5 minutes. Then, negative staining solution was removed from the grids with filter paper, and grids were dried at room temperature. Grids were observed with an H‐7650 transmission electron microscope (Hitachi).

### 2.8 Nanoparticle tracking analysis

Each group of 20 µL EV was diluted 10‐fold with cold PBS so that the particle concentration ranged from 1 to 10 × 10^8^ particles/mL. Nanoparticle tracking analysis (NTA) measurements (NanoSight NS500, NTA 3.2 Dev Build 3.2.16) were used to estimate the size distribution and number of particles in each group of EV.

### 2.9 Cells co‐cultured with ketamine and ketamine‐derived EV

SV‐HUC‐1 cells (1 × 10^5^/well) were seeded in a 12‐well plate and cultured in DMEM/F12 medium supplemented with 12% exosome‐free serum. When the cells reached a 90% confluence, they were treated with different concentrations of ketamine and ketamine‐derived EV. The treatments included the following: A ketamine (0, 1, 2, 4 mmol/L), B ketamine (0, 1, 2, 4 mmol/L) + cytochalasin D, C EV (extracted at 0, 1, 2, 4 mmol/L), D EV (extracted at 0, 1, 2, 4 mmol/L) + cytochalasin D and co‐cultured for 24 hours. Three sub‐holes were provided for each concentration, and 30uL of the liquid volume was added to each well. EV uptake blocker: cytochalasin D (Thermo Fisher) was dissolved in a volume of 30 µL at a concentration of 5 µg/mL.^20^, ^21^

### 2.10 Flow cytometry analysis of oxidative stress levels

Detection of active oxygen was performed using an active oxygen detection kit (Solarbio) according to the manufacturer's instruction. The DCFH‐DA fluorescent probe was diluted with a serum‐free medium to a final concentration of 10 μmol/L at 1:1000. The cell culture medium was removed, and 1 mL of diluted DCFH‐DA was added to a 12‐well plate to fully cover the cells and incubated at 37°C for 20 minutes. Excess DCFH‐DA was removed by washing the cells with serum‐free cell culture thrice. Finally, the CytoFLEX (Beckman Coulter) was used to detect oxidative stress levels in cells, and the FITC channel was selected.

### 2.11 Extracellular vesicle uptake experiment

The EV uptake kit PKH67 (Sigma) was used to determine cellular uptake of fluorescently labelled EV. 20 µL EV were mixed with diluent C according to the manufacturer's instructions. Then, 4 µL PKH67 probe was added, mixed and incubated for 3 minutes at room temperature. The reaction was stopped by adding an equal volume of exosome‐free serum to complete the fluorescent labelling of EV. The PKH67‐labelled EV were then added to a spin column for re‐extraction. The same amount of binding buffer XBP was added and centrifuged at 500 *g* for 1 minute. Then, 8 mL of wash buffer XWP was added and centrifuged at 5000 *g* for 5 minutes. Then, 400 µL of XE eluate was added, and the mixture was incubated for 2 minutes and centrifuged again at 500 *g* for 5 minutes. The mixture was incubated for another 2 minutes and centrifuged at 5000 *g* for 5 minutes. Finally, the isolated EV were added to the cells, and the EV uptake ability of the cells was observed using a fluorescence microscope after 24 hours.

### 2.12 Western blotting analysis

Cell samples or ketamine‐derived EV were lysed in RIPA buffer (Vazyme Biotech) containing protease inhibitors (Beyotime), and the protein concentration was then quantified. Equal amounts of protein (25 µg) were loaded and separated on 12% SDS‐polyacrylamide gels (SDS‐PAGE) and then transferred onto polyvinylidene difluoride (PVDF) membranes (Beyotime). The membranes were incubated with primary antibodies at 4°C overnight and then washed in TBST and incubated with secondary antibody at 37°C for 1 hour. The target band of the proteins was then visualized using HRP substrate (Merck Millipore) and analysed using MD ImageQuant software version 5.2 (Molecular Dynamics, Inc). The primary antibodies used were as follows: CD9 (Abcam), CD81 (SBI), HSP70 (Abcam), Nrf2 (CST, 1:1000), COX‐2 (CST, 1:1000), NF‐kB (CST, 1:1000), p38 (CST, 1:1000), p‐NF‐kB (CST, 1:800) and p‐p38 (CST, 1:800). β‐actin (CST, 1:5000) was used as a loading control.

### 2.13 Real‐time quantitative PCR

 Cells total RNA extraction and reverse transcription PCR: The collected cells were treated with TRIzol reagent (Solebao, R1100), fully lysed and mixed. Thereafter, 50% by volume of chloroform was added, and the upper aqueous phase was collected after centrifugation. An equal volume of isopropanol was added, and RNA was precipitated by centrifugation. The RNA was washed with 70% alcohol, dried and dissolved in RNA‐free enzyme water. Subsequently, RNA concentration and purity were detected using an ultra‐micronucleic acid detector (SuiZheng, FC‐1100), followed by reverse transcription using the reverse transcription kit (Mona, RN05004M) according to the instructions of the kit. 200 ng total RNA was used for cDNA preparation. The resulting cDNA was stored at −20°C until use.

#### 2.13.1 qPCR and data analysis

The qPCR reaction system contained: 5 µL SYBR Green Premix Taq (Mona, RN04006M), 1 µL cDNA (100 ng/µL), 0.3 µL forward primer (10 µmol/L), 0.3 µL reverse primer (10 µmol/L) and 3.4 µL H_2_O. Reaction procedures were as follows: pretreatment at 95°C:30 seconds; PCR cycle (40 cycles): 95°C:5 seconds, 60°C:30 seconds, 72°C:15 seconds; dissolution curve: standard dissolution curve program. The PCR reaction was carried out on the ABI 7500 quantitative PCR instrument. The experimental data were calculated and analysed using SPSS software and analysed by 2^−ΔΔCT^ method. The formula used was as follows: ΔCt = (Ct gene − Ct β‐actin), ΔΔCt = (ΔCt treat − ΔCt control). The relative expression value of a gene was normalized against the expression of Actb from control group (0 µm) mRNA. Primer sequences were given in Table 1.

**Table 1:** Oligonucleotide primer pairs used for real‐time RT‐PCR

Primer name	Sequence	Annealing temperature, °C	Size (bp)	Database
Nrf2‐F	AGGTTGCCCACATTCCCAAA	60.11	118	NM_001145412.3
Nrf2‐R	AGTGACTGAAACGTAGCCGA	59.04		
COX2‐F	GTTCCACCCGCAGTACAGAA	59.97	106	NM_000963.4
COX2‐R	AGGGCTTCAGCATAAAGCGT	60.04		
NF‐kB‐F	CCAACAGATGGCCCATACCT	59.45	174	NM_001165412.1
NF‐kB‐R	AACCTTTGCTGGTCCCACAT	59.81		
p38‐F	TATGCGTCTGACAGGAACACC	60.07	198	NM_001315.2
p38‐R	TGGGCCGCTGTAATTCTCTT	59.38		
ACTB‐F	GTCATTCCAAATATGAGATGCGT	57.32	121	NM_001101.5
ACTB‐R	GCTATCACCTCCCCTGTGTG	59.82		

### 2.14 Luciferase reporter assay

SV‐HUC‐1 cells were seeded at a concentration of 10^6^ cells per well in six‐well plates. Then, they were divided into 4 groups which they were co‐cultured with EV (0 mmol/L), EV (2 mmol/L), EV (0 mmol/L) + inhibitor, and EV (2 mmol/L) + inhibitor, respectively. Our purpose was to verify the effect of EV on cells. We used QNZ (NF‐kB inhibitor, ab141588) and SB203580 (P38 inhibitor, 56335, CST) as inhibitors in groups. After lentivirus infection, pretreated 4 groups of SV‐HUC‐1 cells were transfected with NF‐kB reporter, P38 reporter and negative control reporter for 24 hours following the manufacturer's protocol. Luciferase activity was detected using the Promega Dual Reporter Assay, and relative luciferase activity was calculated as the ratio of firefly (reporter) to Renilla (transfection control) luciferase activity. NF‐kB activity was determined by using the NF‐kB Reporter kit (BPS Bioscience, #60614) while P38 activity was determined by using P38 Reporter kit (Addgene, #52920).

### 2.15 Statistical analysis

All statistical analyses in this study were conducted with GraphPad 8.0 (GraphPad Software Inc), and data were presented as the mean ± SEM. Comparisons between groups were performed using one‐way ANOVA followed by LSD’s post hoc test. *P* < .05 was considered statistically significant.

## 3 RESULTS

### 3.1 High concentrations of ketamine decrease cell vitality

**Figure 1:**
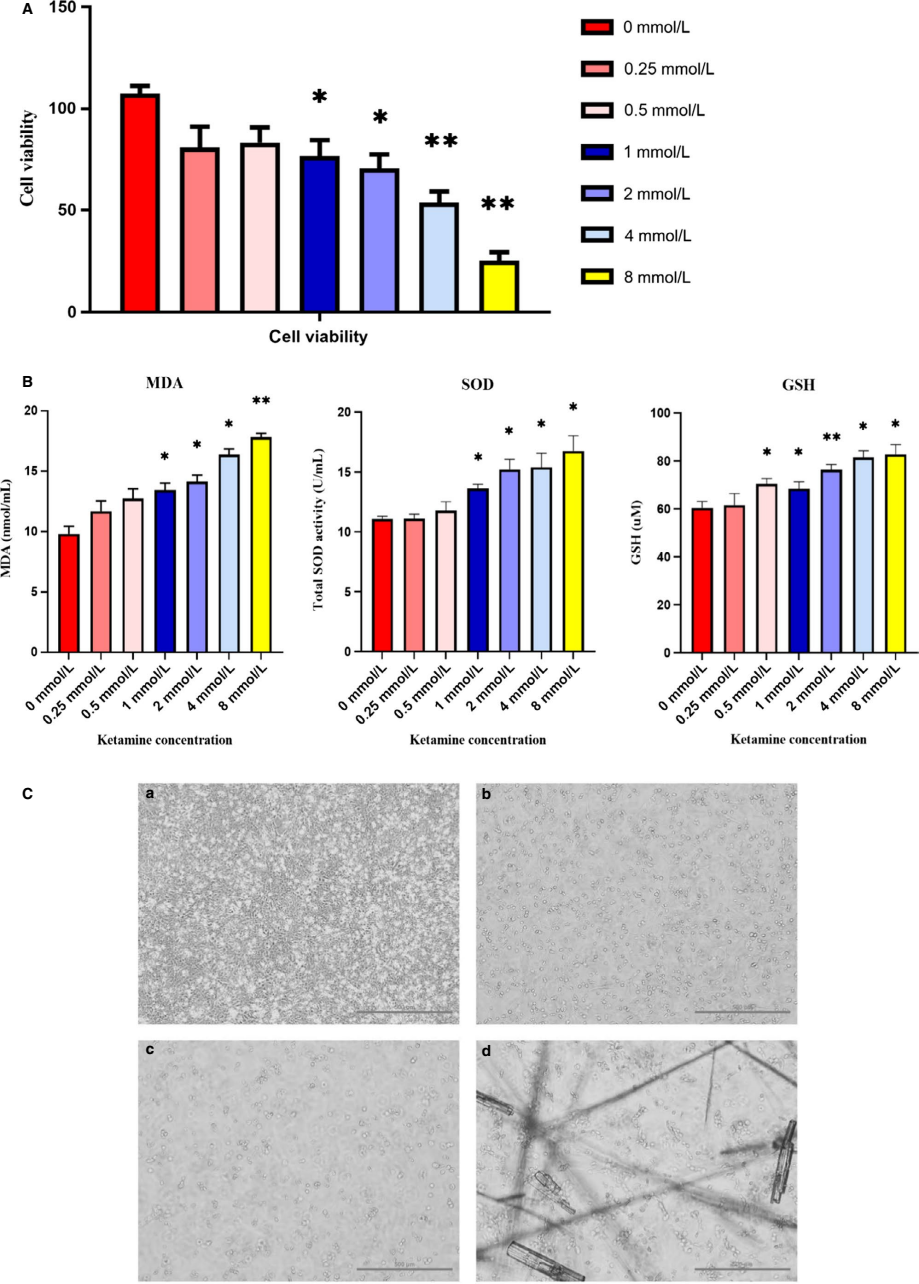
Bladder cytotoxicity and damage caused by ketamine. A, SV‐HUC‐1 cells were exposed to different concentrations (0, 0.25, 0.5, 1, 2, 4 and 8 mmol/L) of ketamine for 48 h. Cell viability was determined by CCK‐8 assay. Ketamine treatment at 0.25 (*P* = .074) or 0.5 (*P* = .052) mmol/L had no effect on cell viability compared to control groups (0 mmol/L). However, when ketamine at a concentration ≥ 1 mmol/L, the cell viability (*P* < .05) was significantly reduced. B, Cell supernatants stimulated with different concentrations of ketamine. Indirect measurement of ROS production by ELISA based on MDA, SOD and GSH levels. C, Cell viability (magnification, 100×) after treatment with 0, 1, 2 and 4 mmol/L ketamine for 48 h. Cells treated with 1 or 2 mmol/L ketamine showed concentrated chromatin and the number and size of the cells decreased. Cells treated with 4 mmol/L Ox showed the appearance of apoptotic bodies and ketamine crystallization. Data were expressed as the mean ± SEM of at least 3 number of biological replicates. Where *P < .05, **P < .01. (a) Cells treated with 0 mmol/L ketamine for 48 h. (b) Cells treated with 1 mmol/L ketamine for 48 h. (c) Cells treated with 2 mmol/L ketamine for 48 h. (d) Cells treated with 4 mmol/L ketamine for 48 h

The Cell Counting Kit‐8 (CCK‐8) assay was used to evaluate cell proliferation and cytotoxicity because it is very sensitive and does not involve the use of radioactive reagents.^22^ Cell damage and cytotoxicity varied among groups but displayed similar trends over time. Comparison between groups showed that ketamine at a concentration of ≥1 mmol/L damaged SV‐HUC‐1 cells in a concentration‐dependent manner (1 mmol/L, *P* = .021; 2 mmol/L, *P* = .019) (Figure 1A). At the same time, when the concentration of ketamine ≥ 4 mmol/L, the cell viability was significantly reduced (4 mmol/L, *P* = .004; 8 mmol/L, *P* = .001).

### 3.2 High concentration of ketamine enhances apoptosis

Our results of the Elisa test showed that enzymatic (SOD) (*P* < .05), non‐enzymatic (GSH) (*P* < .05) antioxidant activity increased in cell supernatants with ketamine concentration ≥ 1 mmol/L. Meanwhile, lipid peroxidation (MDA formation) (*P* < .05) also increased, indicating that oxidative stress up‐regulated in cells (Figure 1B). After 48 hours of exposure to ketamine, apoptosis was increased in ≥1 mmol/L ketamine, which showed nuclear pyknosis, nuclear fragmentation and nuclear lysis (Figure 1C). Cells exposed to 8 mmol/L ketamine showed significant necrosis. Therefore, lower concentrations of ketamine (0, 1, 2 and 4 mmol/L) were used in the subsequent experiments.

### 3.3 Identification of EV

**Figure 2:**
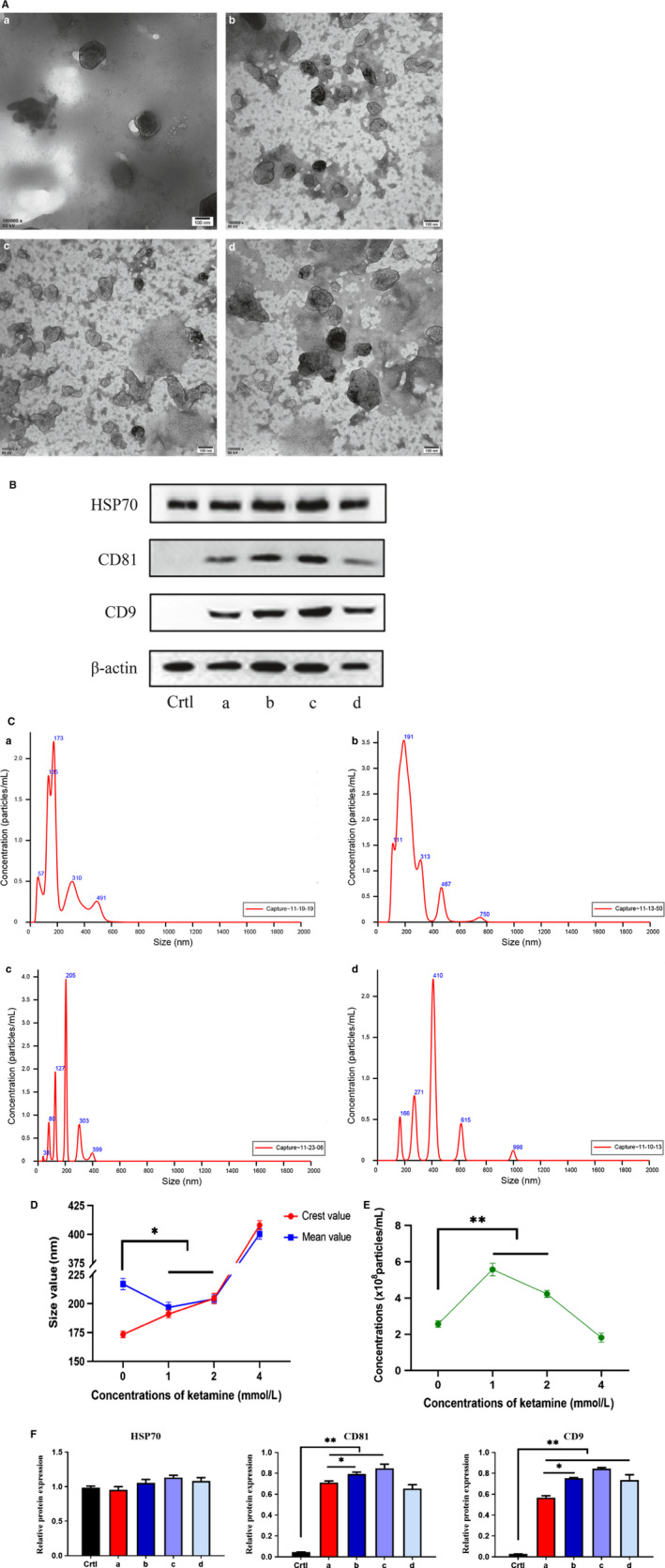
Identification of EV. A, EV observed by an electron microscope. B, Western blot analysis was performed using the EV marker, and the density measurement value of each protein was normalized to the density measurement value of β‐actin. C, NTA particle size detection. D, Size distribution of EV. E, Particle concentration of EV. F, Protein quantification of EV. (ctrl) SV‐HUC‐1 cell samples after EV isolation. (a) Exposure to 0 mmol/L ketamine extracted EV. (b) Exposure to 1 mmol/L ketamine extracted EV. (c) Exposure to 2 mmol/L ketamine extracted EV. (d) Exposure to 4 mmol/L ketamine extracted EV

Electron microscopic images showed that EV derived from SV‐HUC‐1 cells (0, 1, 2, and 4 mmol/L) exhibited the typical membrane vesicle characteristics of cup shape and double‐membrane structure (Figure 2A). Western blot analysis showed that HSP70, CD81 and CD9 were highly expressed in EV.^23^ However, SV‐HUC‐1 cells (after production of EV) expressed HSP70 but did not express either CD81 or CD9 (Figure 2B). The results of nanoparticle tracking analysis (NTA) showed that increasing the concentration of ketamine decreased the average EV size, while increasing EV crest size and concentrations (Figure 2C‐E). The particle concentration of the 0 mmol/L group was 2.58 ± 0.36 × 10^8^ particles/mL, the crest size was 172.9 nm, while the mean size was 217.5 nm. In the 1 mmol/L group, the above values were 5.50 ± 0.29 × 10^8^ particles/mL, 190.4 and 197.6 nm, respectively. The respective values obtained in the 2 mmol/L group were 4.30 ± 0.25 × 10^8^ particles/mL, 204.4 and 203.0 nm. In the 4 mmol/L group, EV concentration was 1.47 ± 0.25 × 10^8^ particles/mL; crest size was 419.2 nm; and mean size was 400.0 nm (Figure S1).

### 3.4 Effects of ketamine concentration on EV protein

As EV are rich in protein, protein quantification can effectively indicate the secretory profiles of EV. The original sample was lysed, and the obtained protein (15 µL/sample) was subjected to Western blot analysis (Figure 2B). Our results indicate that an increase in the concentration of ketamine increased the total protein levels of EV (Figure 2F).

### 3.5 Effects of ketamine group (K) on oxidative stress in SV‐HUC‐1

**Figure 3:**
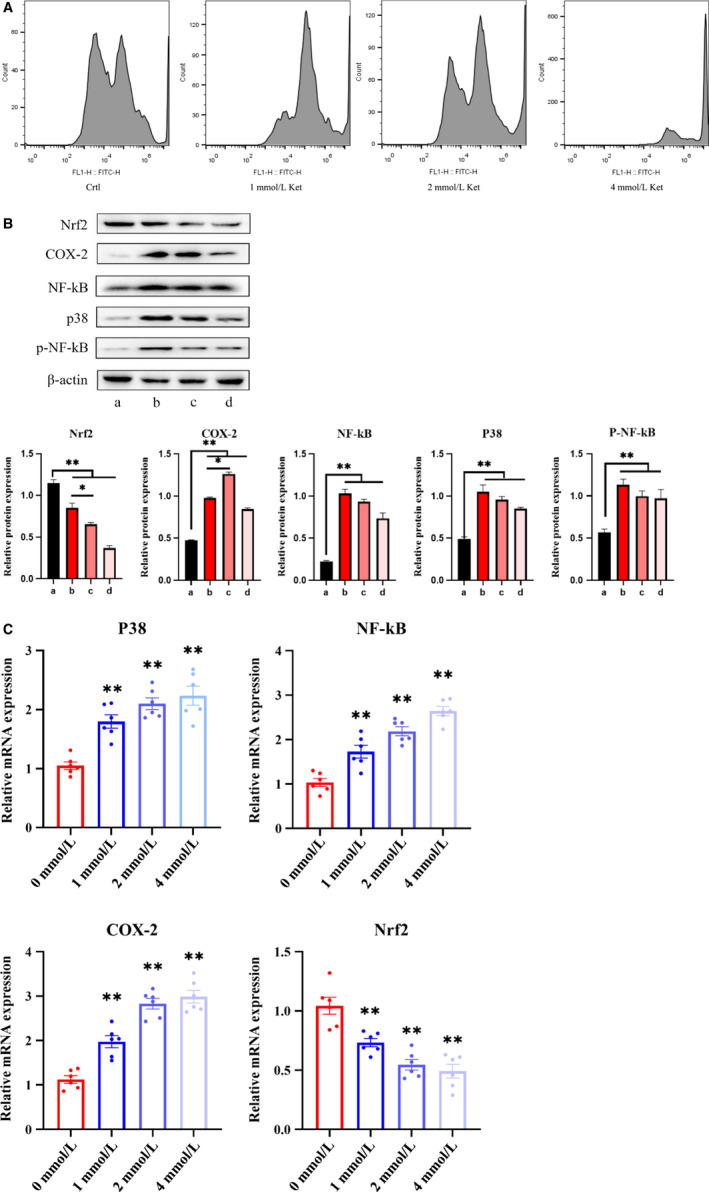
Bladder cells were co‐cultured with ketamine. A, Flow cytometry analysis of oxidative stress. B, Determination of Nrf2, P‐38, Cox‐2, NF‐kB and p‐NF‐kB expression by Western blot in different groups in cells treated with different ketamine concentrations. C, Changes in mRNA expression levels of Nrf2, P‐38, Cox‐2 and NF‐kB after ketamine treatment. All mRNA levels were quantified by quantitative polymerase chain reaction. Data were expressed as the mean ± SEM of at least 3 number of biological replicates.*P < .05, **P < .01. (a), (b), (c) and (d) are similar to Figure 2

Our flow cytometry analysis data confirmed an increase in ketamine concentrations beyond 1 mmol/L results in intracellular ROS levels that are significantly higher than the control group (0 mmol/L), and gradually increased in a concentration‐dependent manner. Among the tested concentrations of ketamine, 4 mmol/L had the highest peak ROS, followed by 2 and 1 mmol/L, while the peak ROS of the control group (0 mmol/L) was at a normal baseline (Figure 3A). The results of a further quantification of inflammatory proteins by Western blot analysis concurred with the above flow cytometry results (Figure 3B). That is, an increase in the concentration of ketamine increases the expression level of cellular inflammatory proteins. At the same time, we found that the expression levels of inflammatory proteins in the 4 mmol/L group were low, which could explain the presence of apoptotic bodies in that group. Cellular necrosis and apoptosis result in the secretion of insufficient inflammatory proteins. Besides, we also determined the relative mRNA expression levels of related proteins by qRT‐PCR (Figure S2), and the results are consistent with the increased production of ROS (Figure 3C).

### 3.6 Effects of ketamine group (K) + cytochalasin D (D) on oxidative stress in SV‐HUC‐1

**Figure 4:**
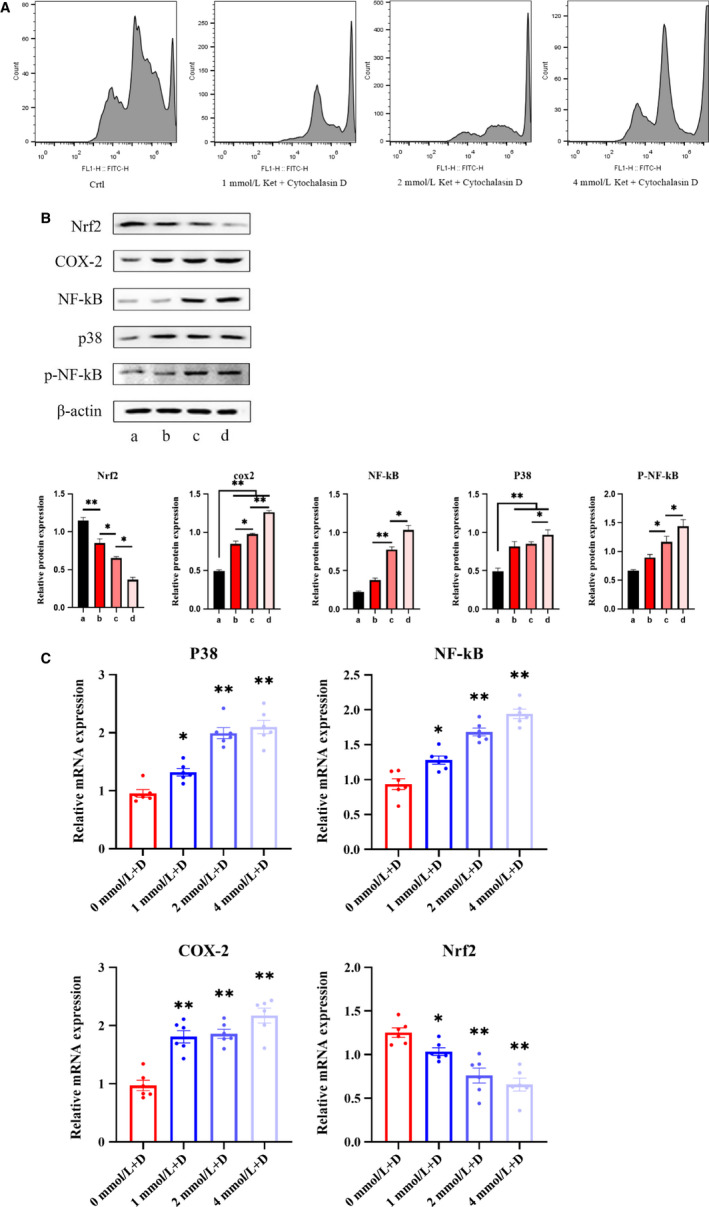
Bladder cells were co‐cultured with ketamine + cytochalasin D. A, Flow cytometry analysis of oxidative stress. B, Determination of protein expression of Nrf2, P‐38, Cox‐2, NF‐kB and p‐NF‐kB in cells. C, Changes in mRNA expression levels of Nrf2, P‐38, Cox‐2 and NF‐kB in cells. Data were expressed as the mean ± SEM of at least 3 number of biological replicates.*P < .05, **P < .01. (a), (b), (c) and (d) are similar to Figure 2

We added cytochalasin D to different concentrations of ketamine‐stimulated SV‐HUC‐1 cells. The results showed (Figure 4A) that cytochalasin D significantly reduced intracellular ROS levels. Specifically, in the K + D group, the (b), (c) and (d) subgroups showed a certain amount of ROS peaks compared with (a) the control group, but they had decreased a lot compared with K group (Figure 3A). In the K + D group, the (a) group was used as the control group, and the baseline was not significantly different from the group (a) in the K group. Western blot analysis (Figure 4B) further showed that after the addition of cytochalasin D, the (d) subgroups in the K + D group showed the highest expression of inflammatory proteins compared with (a‐c), which also decreased a lot (compared with K group). These indicated that although in the K group, the subgroup of (d) caused a decrease in inflammatory protein due to apoptosis. However, because of the addition of EV blocker, apoptosis was relatively reduced and inflammatory proteins were increased again. Besides, the same decrease trend was observed in the expression levels of inflammatory mRNA in the K + D group (Figure 4C).

### 3.7 Effects of extracted EV on oxidative stress in SV‐HUC‐1

**Figure 5:**
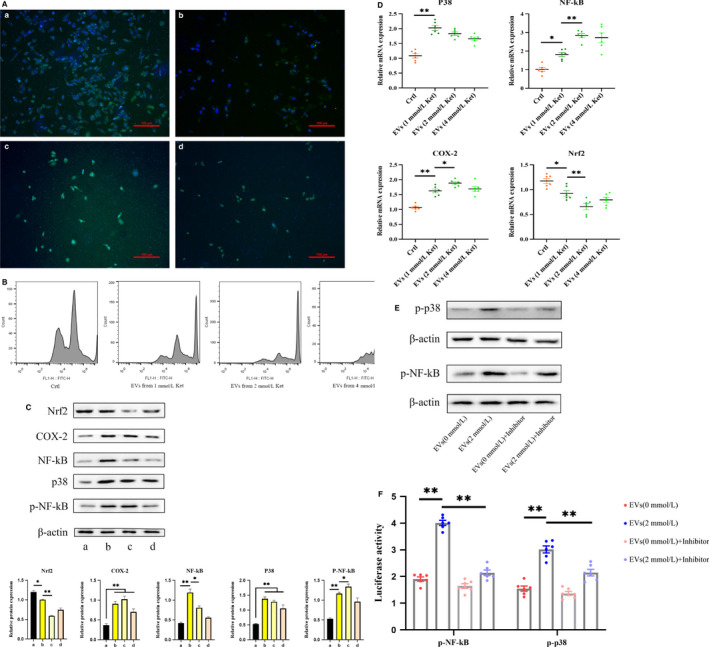
Cells were co‐cultured with ketamine cell‐derived EV. A, Analysis of uptake of ketamine cell‐derived EV by SV‐HUC‐1 cells using confocal microscopy. EV were stained with PKH67 and incubated with cells for 24 h. The nucleus was labelled with DAPI (blue). B, Flow cytometry analysis of oxidative stress. C, Determination of protein expression of Nrf2, P‐38, Cox‐2, NF‐kB and p‐NF‐kB in cells. D, Changes in mRNA expression levels of Nrf2, P‐38, Cox‐2 and NF‐kB in cells. E, Western blotting analysis of phosphorylated p‐NF‐kB, p‐p38 in 4 groups. F, Data of luciferase activity of NF‐kB and P38 promoter in cells, after EV and inhibitor pretreatment. Data were expressed as the mean ± SEM of at least 3 number of biological replicates. *P < .05, **P < .01. (a), (b), (c) and (d) are similar to Figure 2

We further explored the mechanism of action of EV in KIC. The derived EV were stained with PKH67. PKH67‐labelled EV were incubated with bladder epithelial cells (nuclei labelled with DAPI (blue)). After 24 hours, we observed that PKH67‐labelled EV were present in the cell membrane and cytoplasm (Figure 5A). This result indicated that ketamine cell‐derived EV could be incorporated by SV‐HUC‐1 cells, indicating its potential role in regulating bladder epithelial cell function. We also examined the effects of derived EV on bladder epithelial cells. Similarly, flow cytometry (Figure 5B) indicated that EV extracted with ketamine concentration ≥ 1 mmol/L had increased intracellular ROS levels, and the increase occurred in a concentration‐dependent manner. The ROS peak of the 4 mmol/L group EV was the highest. Western blot analysis results (Figure 5C) also indicated that with the increase of ketamine‐induced concentration, the extracted EV also increased the expression level of cellular inflammation. However, the expression of inflammatory proteins was lower in the 4 mmol/L group, probably due to the occurrence of apoptosis, which led to insufficient secretion of EV.

After incubation with the derived EV, we found the phosphorylation level of NF‐kB was significantly increased and the expression of the anti‐oxidative stress transcription factor nuclear factor erythroid‐2–related factor 2 (Nrf2) was decreased. These indicated that ketamine‐induced EV can enhance P38 signal expression and further activate NF‐kB signalling pathway and its phosphorylation level. Also, the downstream molecule COX‐2 expression was up‐regulated. We also used qPCR (Figure 5D) to verify the expression of inflammatory protein factors, and the results were consistent with the above.

As we know that NF‐kB and P38 are biologically activated after they are phosphorylated, it is important to investigate whether EV affect inflammation occurs through the phosphorylation of NF‐kB or P38 signalling. To this end, QNZ and SB203580 were used to directly inhibit NF‐kB and P38 signalling (Figure S3). Western blotting analysis indicated that the inhibitor dramatically decreased the expression of phosphorylated NF‐kB and P38 in cells (Figure 5E). We then performed a luciferase reporter assay with constructs driven by a human NF‐kB and P38 promoter. As expected, inhibitor led to a significant transient expression decrease of NF‐kB and P38 promoter activity in EV‐induced cells (Figure 5F). Interestingly, we also found that EV (2 mmol/L) group had higher NF‐kB and P38 luciferase activity and significantly higher phosphorylation levels than EV (0 mmol/L) group.

Altogether, these data suggest that ketamine cell‐derived EV played significant roles in KIC.

### 3.8 Effects of extracted EV + cytochalasin D on oxidative stress in SV‐HUC‐1

**Figure 6:**
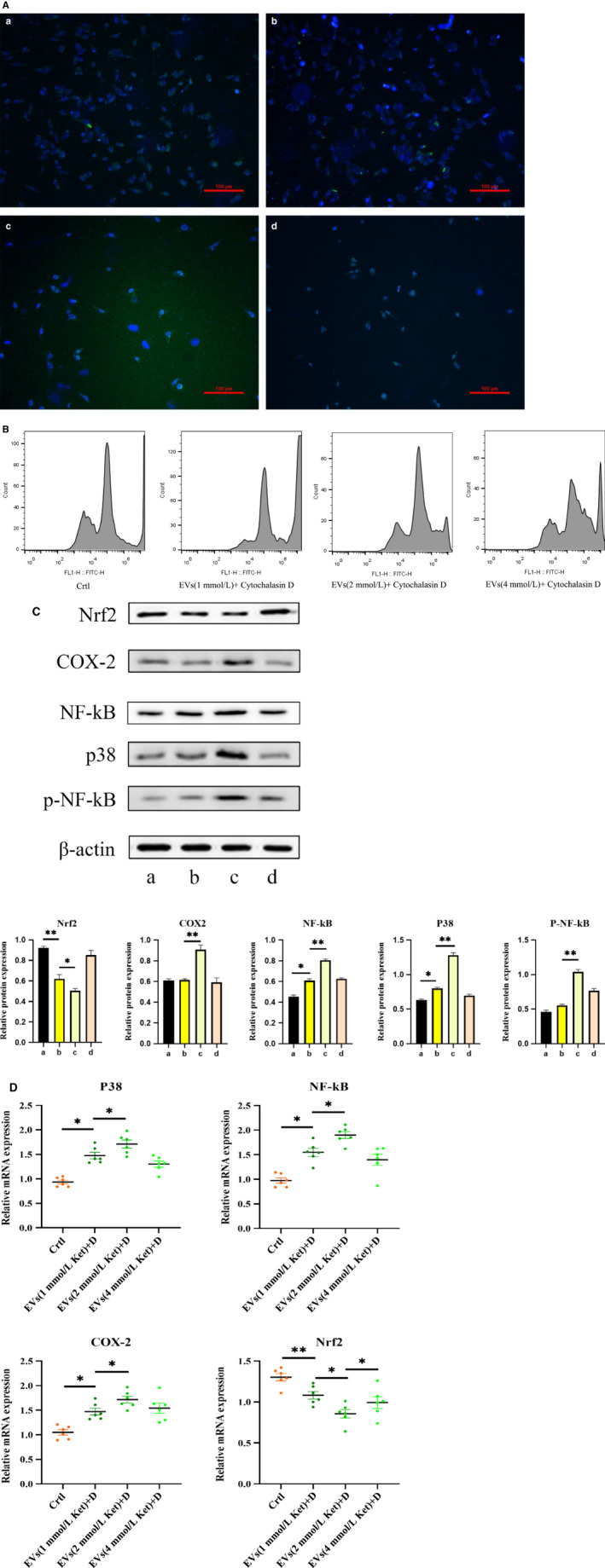
Cells were co‐cultured with ketamine cell‐derived + cytochalasin D. A, Inhibition of EV uptake. B, Flow cytometry analysis of oxidative stress. B, Determination of protein expression of Nrf2, P‐38, Cox‐2, NF‐kB and p‐NF‐kB in cells. C, Changes in mRNA expression levels of Nrf2, P‐38, Cox‐2 and NF‐kB in cells. Data were expressed as the mean ± SEM of at least 3 number of biological replicates.*P < .05, **P < .01. (a), (b), (c) and (d) are similar to Figure 2

We then co‐cultured the extracellular vesicle blocker (cytochalasin D) with EV and cells for 24 hours. It was found that in the presence of cytochalasin D, the passage of PKH67‐labelled EV through the cell membrane was blocked, although some EV entered the cytoplasm (Figure 6A).

Similarly, we determined the effect of derivatized EV + cytochalasin D on bladder epithelial cells. The flow cytometry data (Figure 6B) indicated that the addition of cytochalasin D to the same concentration of EV resulted in a significant decrease in the ROS peak. In the EV + D group, (b), (c), (d), compared with the (a) control group, although the expression of inflammatory protein (Figure 6C) and mRNA (Figure 6D) was high, their expression levels had decreased a lot following addition of cytochalasin D. Phosphorylation of NF‐kB was reversed and returned to the original baseline level due to the presence of cytochalasin D. The upstream P38 pathway was inhibited, and the expression of the downstream molecule COX‐2 was also significantly reduced. However, the expression of antioxidant transcription factor Nrf2 was increased.

## 4 DISCUSSION

SV‐HUC‐1 cells exhibit oxidative stress damage after exposure to high concentrations of ketamine. Studies have shown that oxidative stress damage affects miRNAs in bladder epithelial cells and activates some pathways involved in the formation of interstitial cystitis.^24^, ^25^ Subsequent changes in miRNA expression may affect the secretion of bladder EV.^10^ For example, EV secreted into the urine of patients with Hunner‐type interstitial cystitis (HIC) show up‐regulation of MEG3 levels. At the same time, MEG3 down‐regulated the expression of miR‐19a‐3p. These markers are highly specific for HIC.^11^ Therefore, we hypothesize that ketamine‐injured SV‐HUC‐1 cells may also secrete EV carrying specific miRNAs.

Successful isolation of EV is one of the key achievements of this study. As this study requires a large number of EV for subsequent experiments, a relatively simple but robust protocol for isolation of high concentrations and yield of EV should be established. The current mainstream EV extraction protocol is ultracentrifugation. Although the method has lower cost, the extracted EV have higher purity but the operation is complicated and time‐consuming, and the loss of EV is enormous.^26^ We, therefore, used the exoEasy Maxi Kit (QIAGEN, Germany) to isolate ketamine‐derived EV.^27^ The elution of intact vesicles from the exoEasy membrane and the yield of EV were better than ultracentrifugation, which is particularly beneficial for subsequent studies of EV function.^28^

Our results indicate that ketamine concentration affects the size and secretion of EV. The four EV subtypes may carry different signalling molecules and play different roles in a given inflammatory microenvironment.^29^ EV are secreted by fusion with a membrane and secreted into the extracellular environment by exocytosis. These endogenous nanocarriers typically carry biologically active molecules, such as RNA and proteins, which are stably present in the extracellular environment and regulate the metabolic pathways of target cells.^30^

Moreover, the EV secreted by SV‐HUC‐1 cells in various environments vary greatly in shape, size distribution and secretion rate. Previous studies have reported that a more limited particle size distribution is associated with faster absorption of target cells and greater efficacy in the microenvironment.^31^ Therefore, we can assume that different concentrations of EV subtype trigger different signal molecules. For example, cells exposed to high concentrations of ketamine secrete many similarly sized exosomes, which facilitates the absorption of target cells. Our results showed that higher concentrations of ketamine damage SV‐HUC‐1 cells, inducing donor cells to secrete numerous exosomes. We considered that EV act as a ‘messenger’ in high‐concentration ketamine environments, and we planned to further understand these signalling molecules by sequencing the EV proteins.

To verify the above conjecture, we did an EV function test. Most experimental evidence suggests that EV usually enter the endosomal compartment^21^, ^32^ by endocytosis. Ingestion can be very rapid, and EV can be found in cells as early as 15 minutes after initial introduction. Cytochalasin D is a metabolite known to depolymerize the actin filament network, leading to inhibition of the endocytic pathway.^33^ Cytochalasin D treatment has been shown, on several occasions in various cell types, to significantly reduce, but not entirely prevent, EV uptake in a dose‐dependent manner.^32^, ^34^ Our results showed that the addition of cytochalasin D confined most of the EV to the extracellular space, but some EV could still enter the cytoplasm and cannot be completely eliminated. This finding could be explained by postulating that EV uptake occurs through more than one mechanism.^35^

Current research suggests that oxidative stress occurs when ketamine and its metabolites are toxic to the bladder urinary tract.^2^ An increase in ROS generation and reduced clearance might be vital to the pathogenesis of urinary system damage caused by certain chemicals and poisons.^36^ The P38 signal is the most crucial member of the MAPK family that controls inflammatory responses, which can be activated by inflammatory stress to participate in apoptosis. Activation of P38 causes nuclear translocation and phosphorylation of many protein kinases and transcription factors including NF‐kB. Activation of NF‐kB plays a crucial role in the development of various inflammatory diseases. A number of studies have shown that NF‐kB plays an important role in KIC. For example, the study by Lee et al^37^ revealed that hyaluronan perfusion treatment significantly inhibits the activation of the NF‐kB signalling pathway through anti‐oxidative‐nitrosation stress and pro‐inflammatory cytokine production. The Chuang Shu Mien team also found epigenetic regulation of COX‐2 expression by DNA hypomethylation via NF‐kB activation in ketamine‐induced ulcerative cystitis.^38^ In other studies, carbesolone could treat interstitial cystitis caused by cyclophosphamide, mainly by inhibiting P38 activation and reducing cyclophosphamide‐induced bladder oxidative stress damage.^39^ Nrf2 is a crucial transcription factor regulating cellular oxidative stress, which can alleviate cellular damage caused by reactive oxygen species and electrophiles and maintain the body's redox homeostasis.^40^

In summary, we first extracted EV secreted by SV‐HUC‐1 cells exposed to high concentrations of ketamine, using a new protocol to distinguish the four subtypes of EV. Our study demonstrated an important mechanism in the development of KIC. That is, ketamine cell‐derived EV mediate urinary epithelial cell‐to‐cell communication and enhance oxidative stress in ketamine‐induced cystitis probably via the P38/NF‐kB pathway. We have reason to believe that this study opens up new horizons for studying the mechanism of KIC formation.

## CONFLICT OF INTEREST

The authors have no competing interest to declare.

## AUTHORS' CONTRIBUTIONS

Xiao jian Xi, Jin jiang Zeng and Yong Lu designed this study. Xiao jian Xi and Shao hua Chen carried out experiments. Zhi wen Jiang and Peng jie He took on the statistical analysis. Xiao jian Xi and Hua Mi drafted the manuscript and revised the manuscript. Hua Mi provided experimental funds and experimental platforms. All the authors had the approval of the submitted and published versions.

## Supporting information

Supplementary MaterialEV

Supplementary Material

Supplementary Material

## Data Availability

The data used to support findings of the study are available from the corresponding author upon reasonable request.
