# Supplementary material for: Extracellular vesicles enhance oxidative stress through P38/NF‐kB pathway in ketamine‐induced ulcerative cystitis
Source: J Cell Mol Med. 2020 May 22;24(13):7609–24. doi: 10.1111/jcmm.15397 (PMC7339200; doi:10.1111/jcmm.15397)

2020-2-7 p-NF-kB and p-p38 WB.


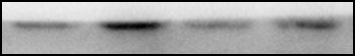

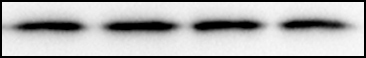

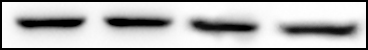

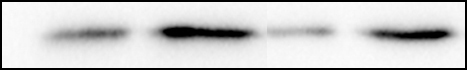


2020-2-10 luciferase activity of NF-kB and P38 in cells co-cultured with EVs.

NF-kB Plasmid


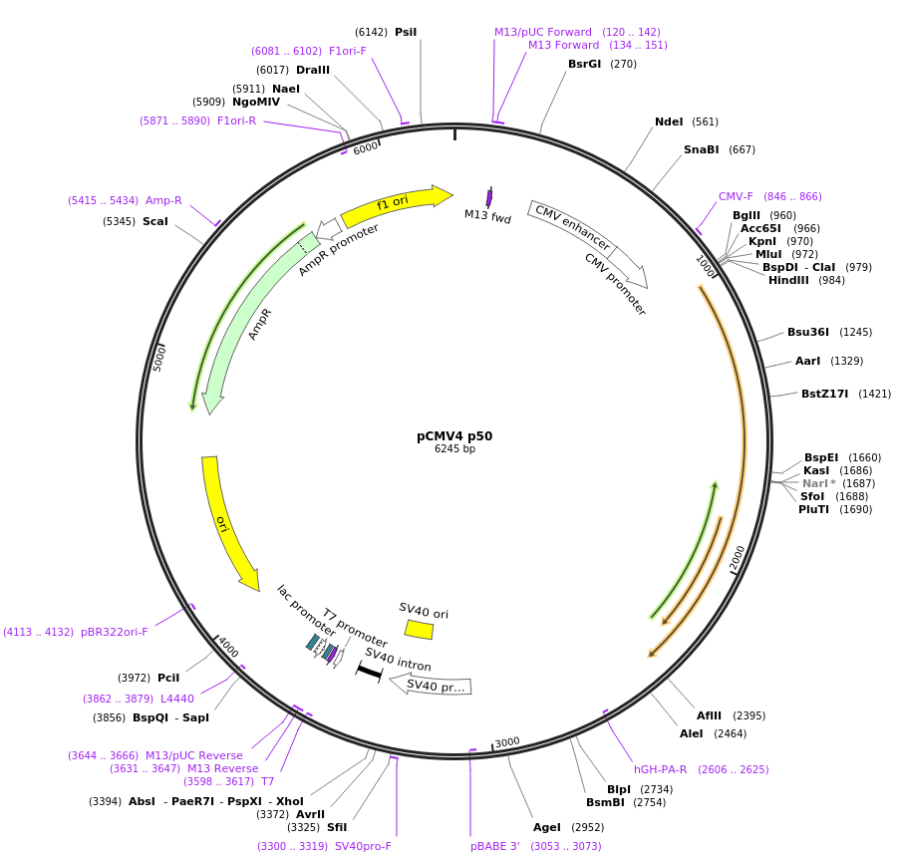


P38 Plasmid


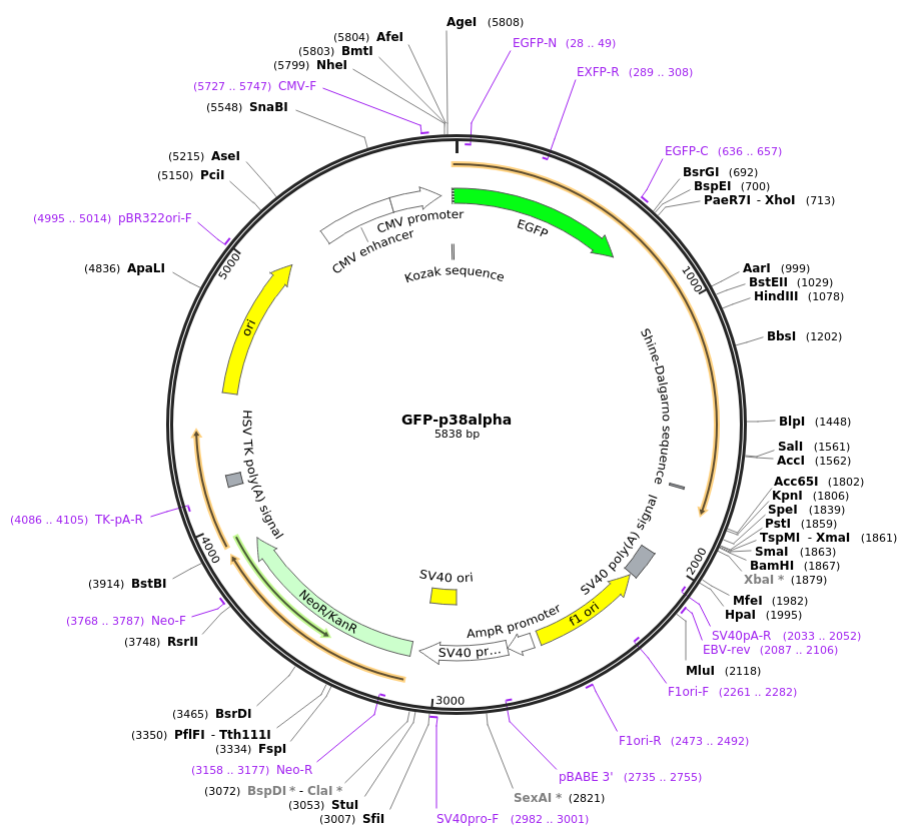

Supplement: Supplementary file 1 — Supplementary MaterialEV [file JCMM-24-7609-s001.docx]
