# Supplementary figures and images for: Extracellular vesicles enhance oxidative stress through P38/NF‐kB pathway in ketamine‐induced ulcerative cystitis
Source: J Cell Mol Med. 2020 May 22;24(13):7609–24. doi: 10.1111/jcmm.15397 (PMC7339200; doi:10.1111/jcmm.15397)

S (1-1)
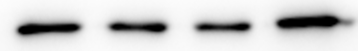


(1-2)
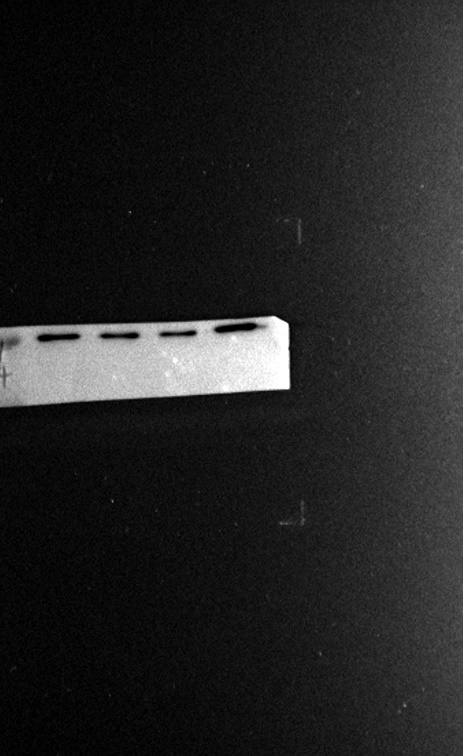


(1-3)



S (2-1)
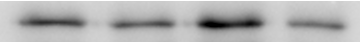


(2-2)



(2-3)
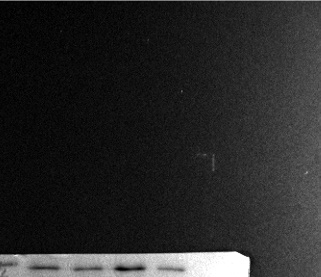


S (3-1)
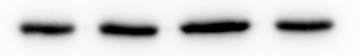


(3-2)
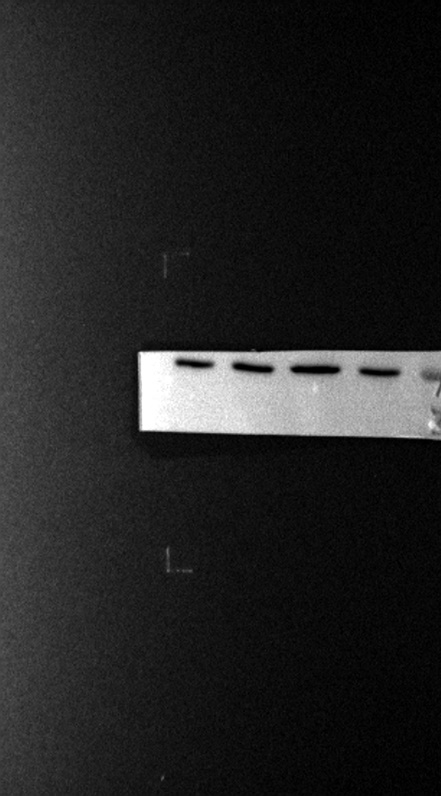


(3-3)



S(4-1)
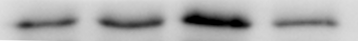


(4-2)
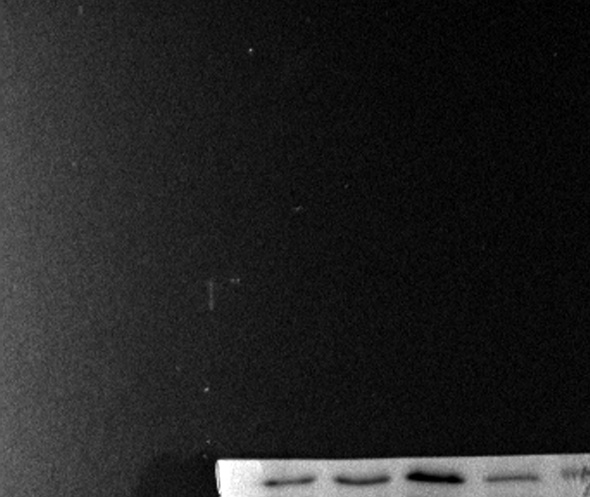


(4-3)



S(5-1)
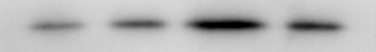


(5-2)



(5-3)
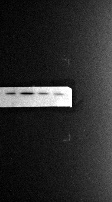


S (6)


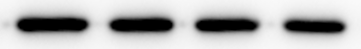


S(7-1)
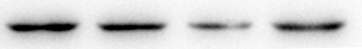


7-2
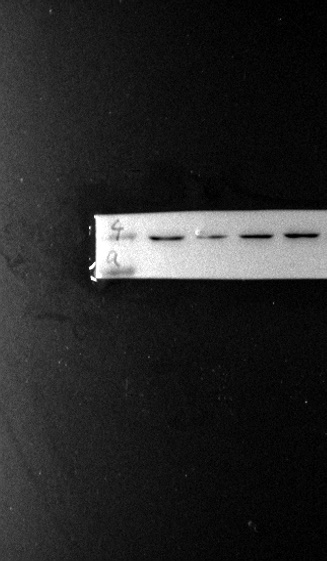


7-3



S(8-1)
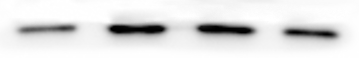


8-2



8-3
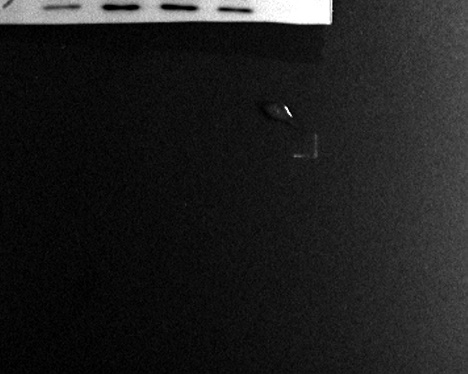


S(9-1)
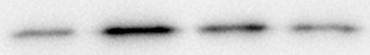


9-2
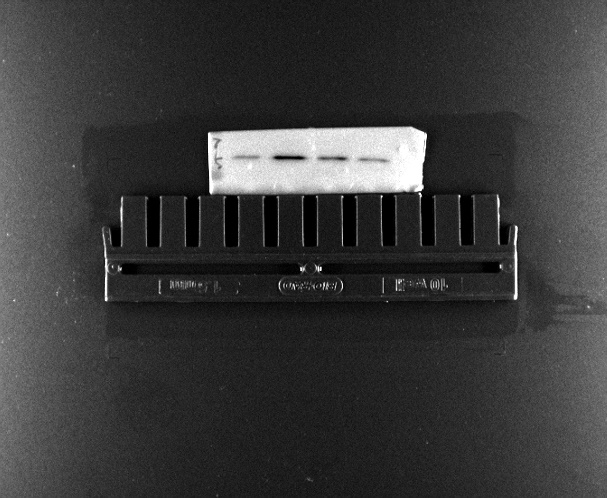


9-3



S(10-1)
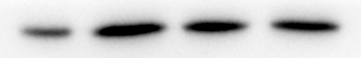


10-2



10-3
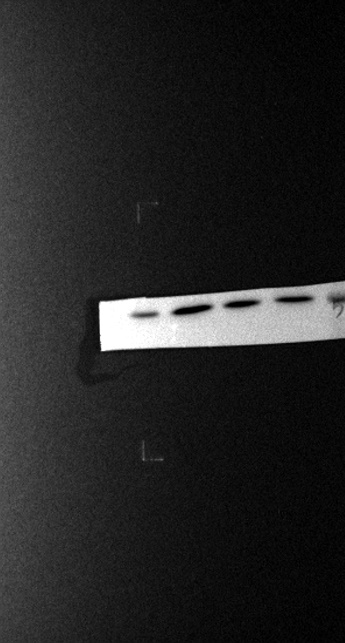


S(11-1)
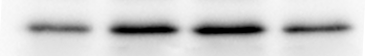


11-2
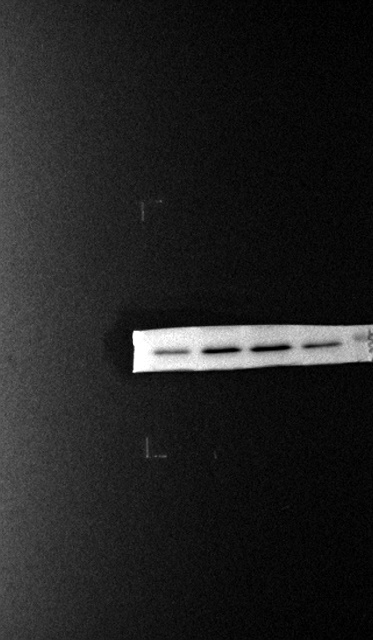


11-3



S(12-1)
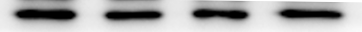


S(13-1)
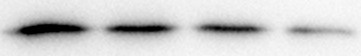


13-2
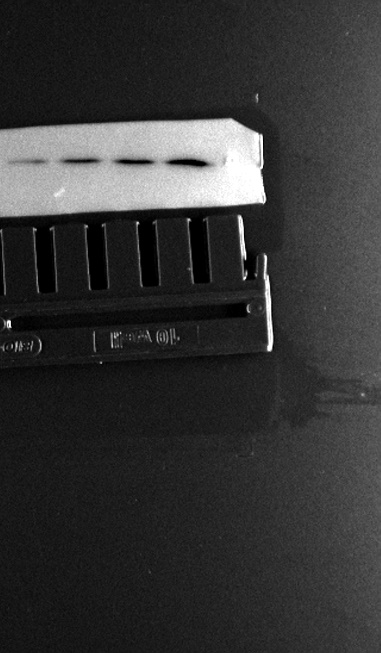


13-3



S(14-1)
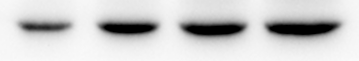


14-2
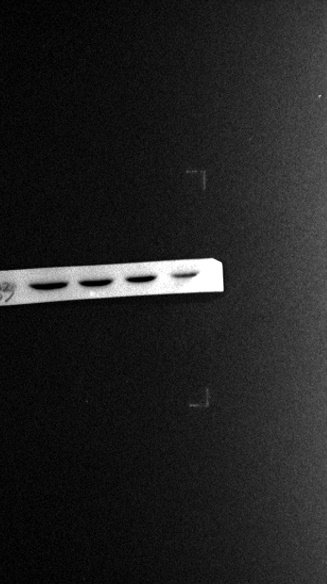


14-3



S(15-1)
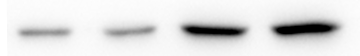


15-2
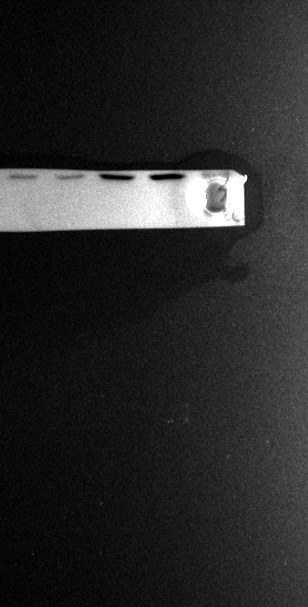


15-3



S(16-1)
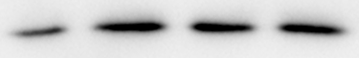


16-2
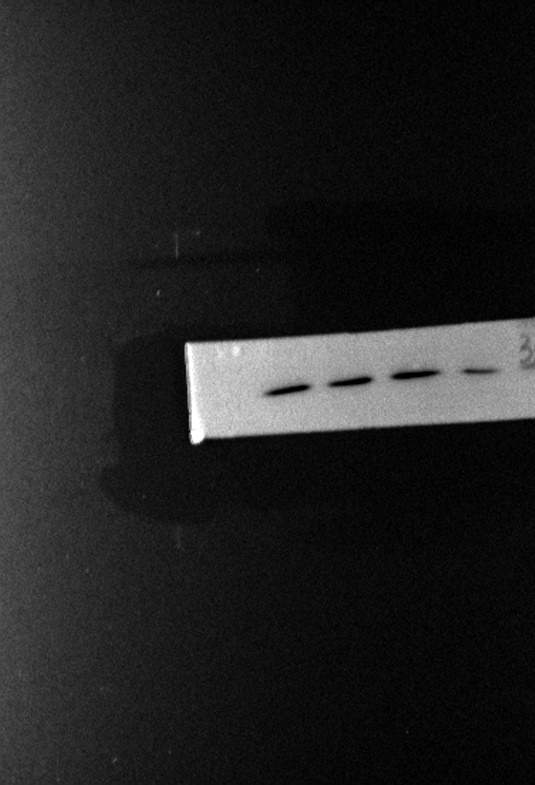


16-3



S(17-1)
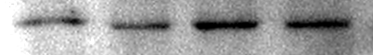


17-2
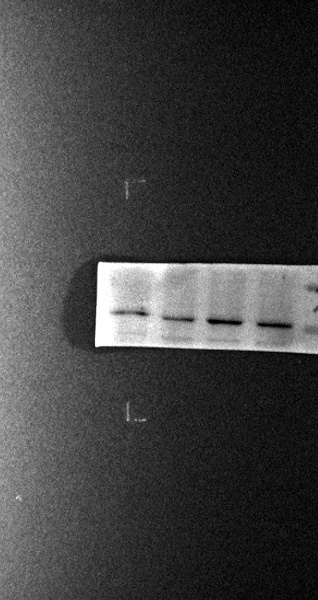


17-3



S(18-1)
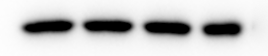


S(19-1)
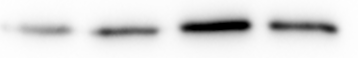


19-2
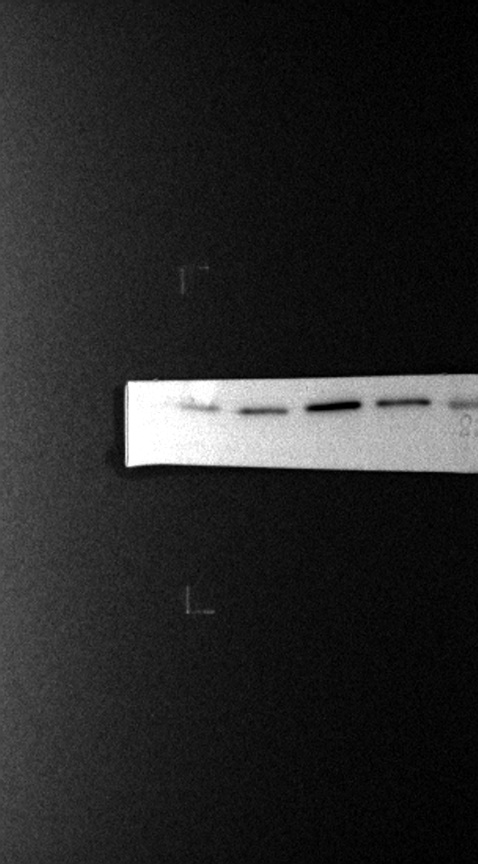


19-3



S(20-1)
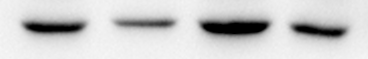


20-2



20-3
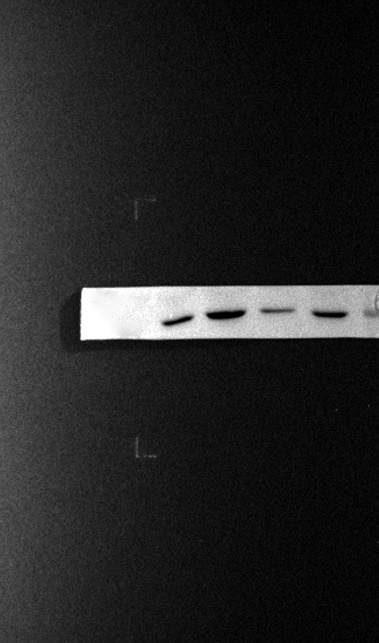


S(21-1)
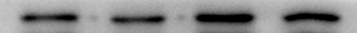


21-2
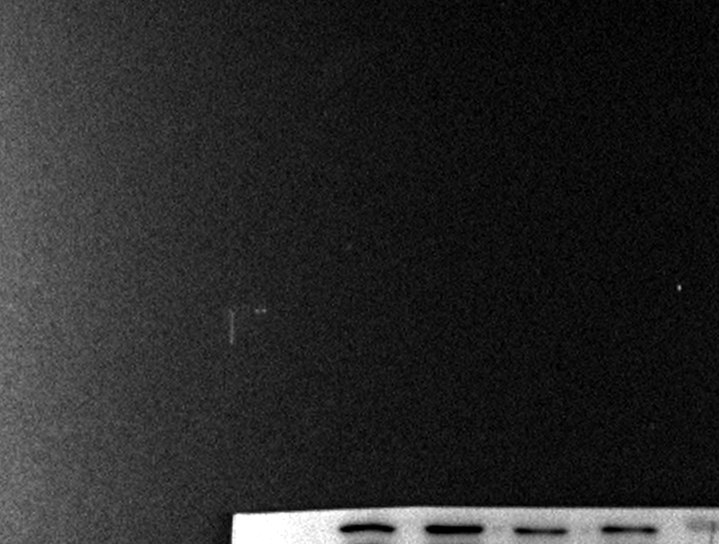


21-3
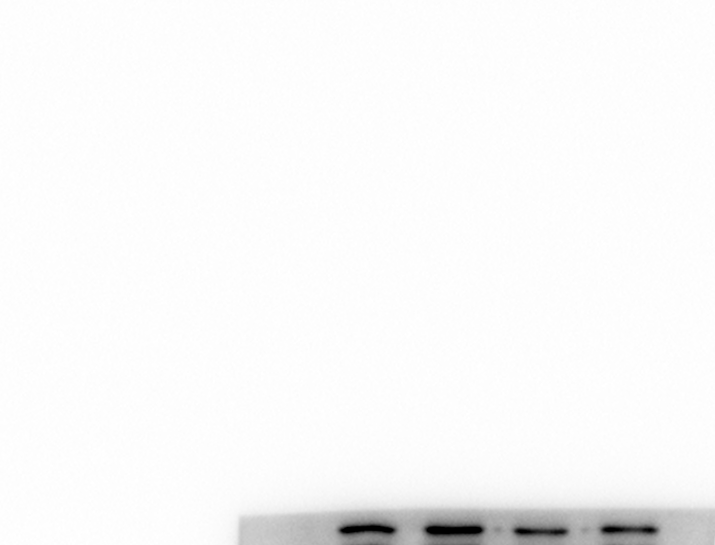


S(22-1)
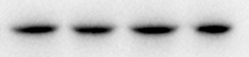


22-2
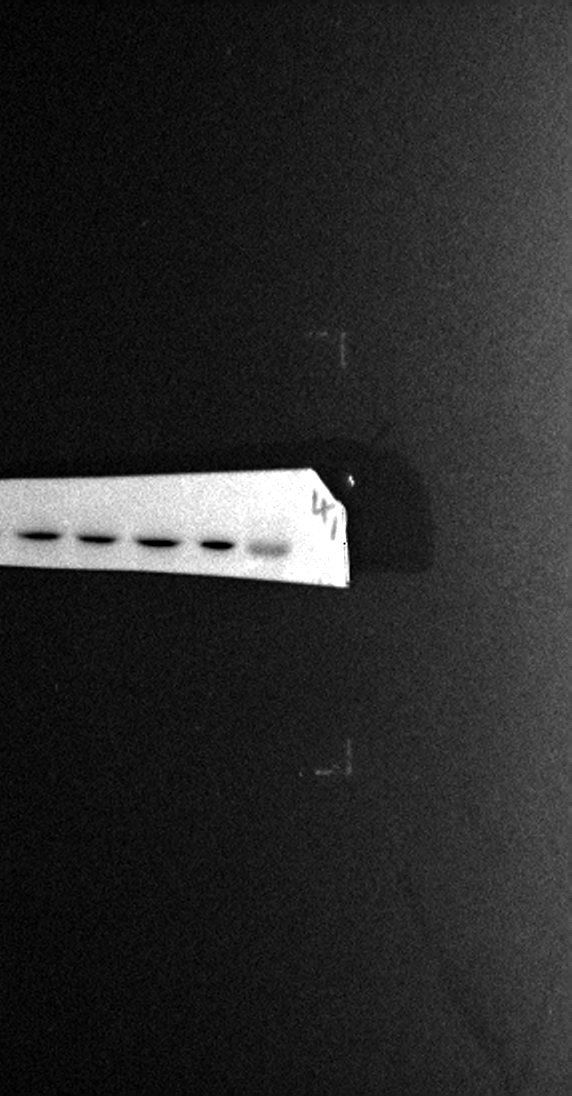


22-3
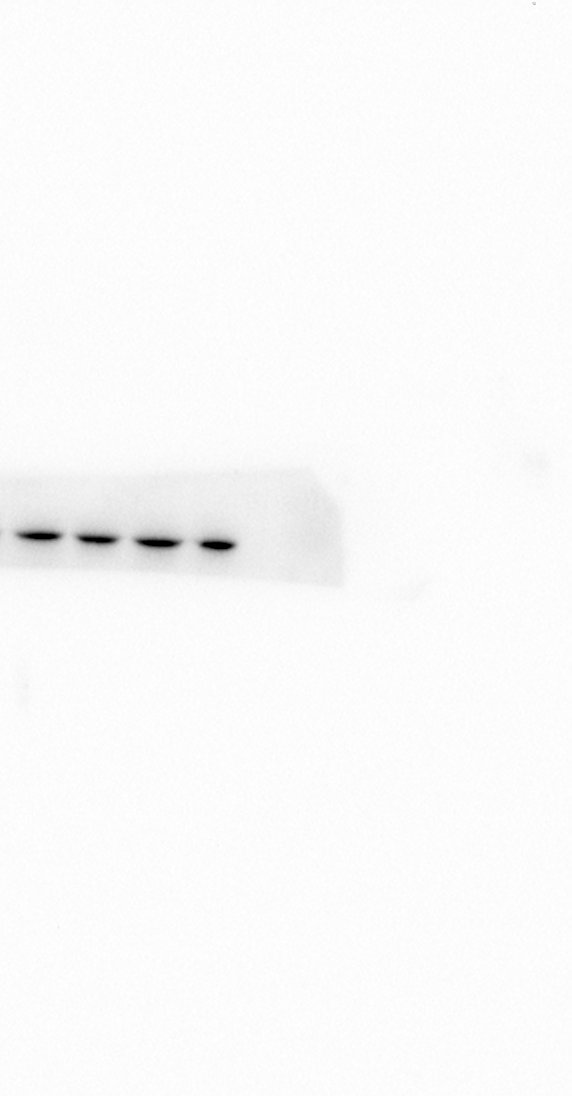


S(23-1)
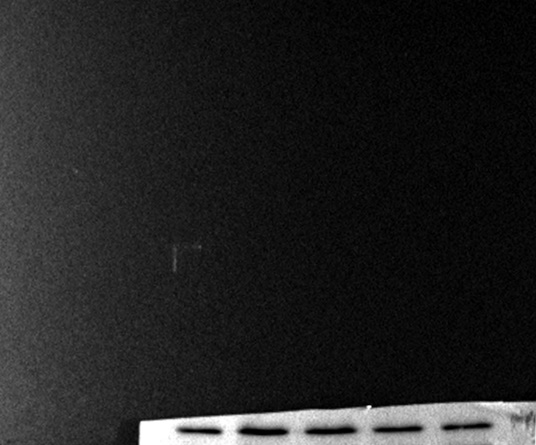


23-2



23-3
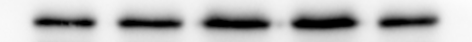


S(24-1)
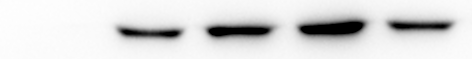


24-2
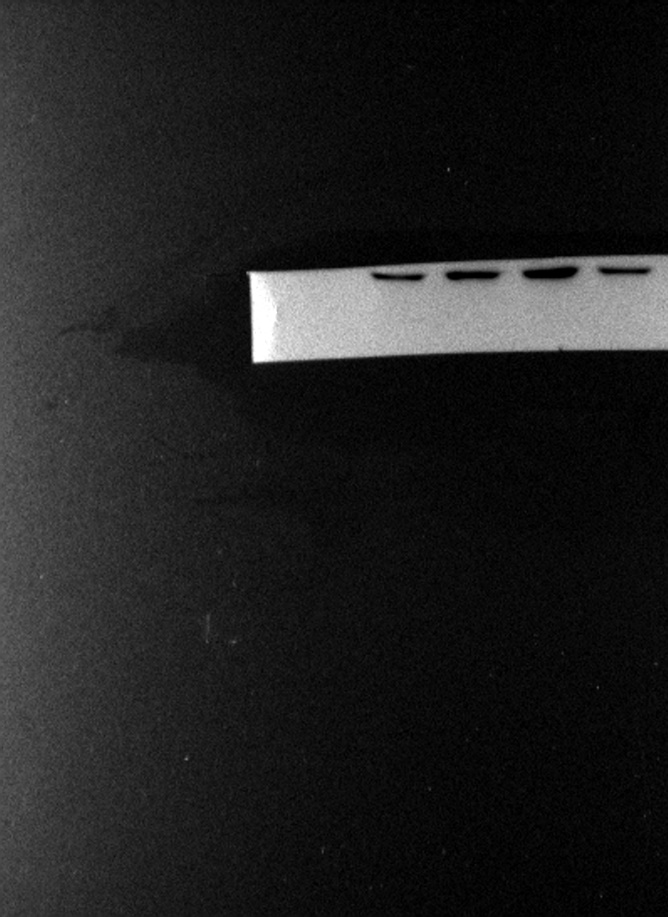


24-3



S(25-1)
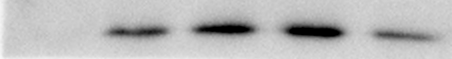


25-2



25-3
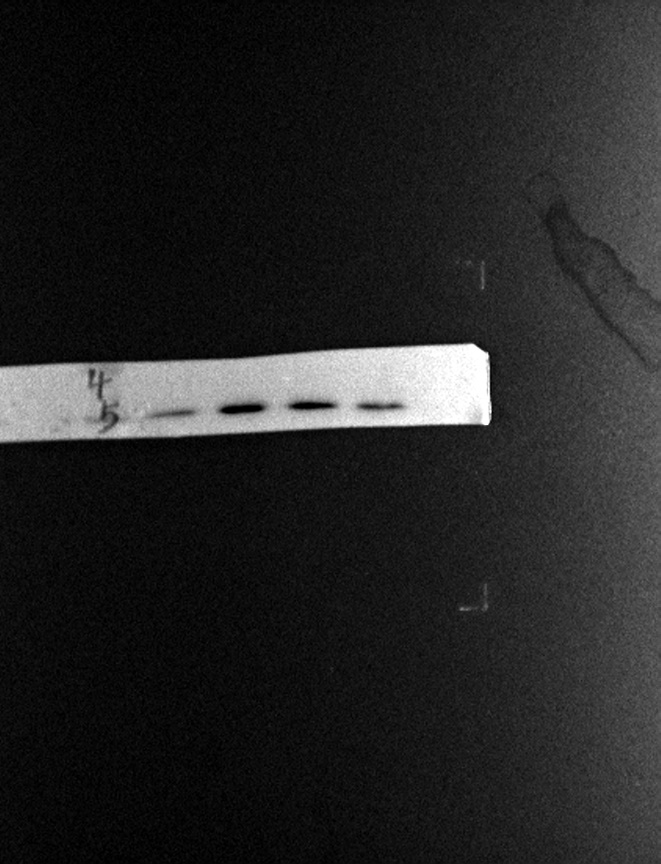


S(26-1)
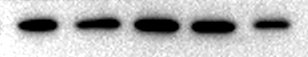


26-2
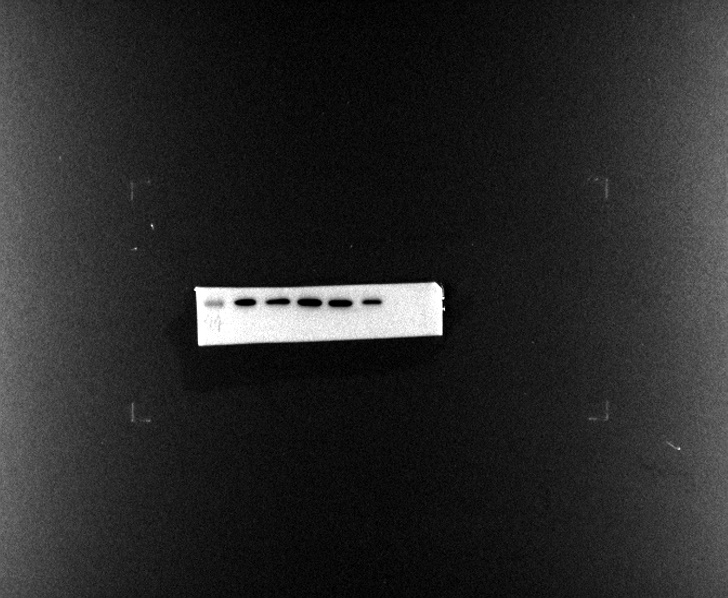

Supplement: Supplementary file 2 — Supplementary Material [file JCMM-24-7609-s002.docx]
