# Supplementary material for: Extracellular vesicles enhance oxidative stress through P38/NF‐kB pathway in ketamine‐induced ulcerative cystitis
Source: J Cell Mol Med. 2020 May 22;24(13):7609–24. doi: 10.1111/jcmm.15397 (PMC7339200; doi:10.1111/jcmm.15397)
Supplement: Supplementary file 3 — Supplementary Material [file JCMM-24-7609-s003.docx]

| S27 |  | **目标基因** | **(内参)b-atcin** | **△CT** | **标准参比** | **△△CT** | **—△△CT** | 2—△△CT | 平均值 |
| --- | --- | --- | --- | --- | --- | --- | --- | --- | --- |
| NFKB | 1 | 25.95372963 | 12.93815231 | 13.01557732 | 12.5696309 | 0.445946376 | -0.445946376 | 0.73 | 1.02 |
|  |  | 25.21609497 | 12.95792007 | 12.2581749 | 12.5696309 | -0.311456045 | 0.311456045 | 1.24 |  |
|  |  | 25.3065834 | 12.87144279 | 12.43514061 | 12.5696309 | -0.134490331 | 0.134490331 | 1.10 |  |
|  | 2 | 25.1354332 | 14.09781075 | 11.03762245 | 12.5696309 | -1.532008489 | 1.532008489 | 2.89 | 3.15 |
|  |  | 25.02239227 | 14.00054359 | 11.02184868 | 12.5696309 | -1.547782262 | 1.547782262 | 2.92 |  |
|  |  | 24.90138435 | 14.19437695 | 10.70700741 | 12.5696309 | -1.862623533 | 1.862623533 | 3.64 |  |
|  | 3 | 25.47964745 | 13.98803425 | 11.4916132 | 12.5696309 | -1.078017743 | 1.078017743 | 2.11 | 2.41 |
|  |  | 25.2857254 | 14.16033268 | 11.12539272 | 12.5696309 | -1.444238218 | 1.444238218 | 2.72 |  |
|  |  | 25.4681633 | 14.15348434 | 11.31467896 | 12.5696309 | -1.254951986 | 1.254951986 | 2.39 |  |
|  | 4 | 25.06818619 | 13.97717381 | 11.09101238 | 12.5696309 | -1.478618558 | 1.478618558 | 2.79 | 3.08 |
|  |  | 25.08081436 | 14.13511276 | 10.9457016 | 12.5696309 | -1.623929342 | 1.623929342 | 3.08 |  |
|  |  | 24.90414886 | 14.09090328 | 10.81324558 | 12.5696309 | -1.756385358 | 1.756385358 | 3.38 |  |
|  | 5 | 25.4740078 | 14.15561962 | 11.31838818 | 12.5696309 | -1.251242765 | 1.251242765 | 2.38 | 2.41 |
|  |  | 25.52550735 | 14.20073509 | 11.32477226 | 12.5696309 | -1.244858678 | 1.244858678 | 2.37 |  |
|  |  | 25.47517052 | 14.21011925 | 11.26505127 | 12.5696309 | -1.304579671 | 1.304579671 | 2.47 |  |
|  | 6 | 25.85852013 | 14.23030663 | 11.6282135 | 12.5696309 | -0.94141744 | 0.94141744 | 1.92 | 1.91 |
|  |  | 25.83578262 | 14.13459301 | 11.70118961 | 12.5696309 | -0.868441327 | 0.868441327 | 1.83 |  |
|  |  | 25.76151085 | 14.17999363 | 11.58151722 | 12.5696309 | -0.988113721 | 0.988113721 | 1.98 |  |
|  | 7 | 25.76245499 | 14.19079304 | 11.57166195 | 12.5696309 | -0.997968992 | 0.997968992 | 2.00 | 1.88 |
|  |  | 25.83661575 | 14.06950092 | 11.76711483 | 12.5696309 | -0.802516111 | 0.802516111 | 1.74 |  |
|  |  | 25.82397957 | 14.17508602 | 11.64889355 | 12.5696309 | -0.920737394 | 0.920737394 | 1.89 |  |
|  | 8 | 26.94579124 | 13.28041649 | 13.66537476 | 12.5696309 | 1.095743815 | -1.095743815 | 0.47 | 0.54 |
|  |  | 26.44377899 | 13.28260231 | 13.16117668 | 12.5696309 | 0.591545741 | -0.591545741 | 0.66 |  |
|  |  | 26.82400703 | 13.24913788 | 13.57486916 | 12.5696309 | 1.005238215 | -1.005238215 | 0.50 |  |
|  |  |  |  |  |  |  |  |  |  |
| C0X | 1 | 21.39418602 | 12.93815231 | 8.456033707 | 8.34454664 | 0.111487071 | -0.111487071 | 0.93 | 1.00 |
|  |  | 21.26865768 | 12.95792007 | 8.31073761 | 8.34454664 | -0.033809026 | 0.033809026 | 1.02 |  |
|  |  | 21.13831139 | 12.87144279 | 8.266868591 | 8.34454664 | -0.077678045 | 0.077678045 | 1.06 |  |
|  | 2 | 21.09426308 | 14.09781075 | 6.996452332 | 8.34454664 | -1.348094304 | 1.348094304 | 2.55 | 2.50 |
|  |  | 21.17316055 | 14.00054359 | 7.172616959 | 8.34454664 | -1.171929677 | 1.171929677 | 2.25 |  |
|  |  | 21.09866905 | 14.19437695 | 6.904292107 | 8.34454664 | -1.440254529 | 1.440254529 | 2.71 |  |
|  | 3 | 21.2341568 | 13.98803425 | 7.246122551 | 8.34454664 | -1.098424085 | 1.098424085 | 2.14 | 2.24 |
|  |  | 21.32005806 | 14.16033268 | 7.15972538 | 8.34454664 | -1.184821256 | 1.184821256 | 2.27 |  |
|  |  | 21.29846115 | 14.15348434 | 7.144976807 | 8.34454664 | -1.199569829 | 1.199569829 | 2.30 |  |
|  | 4 | 21.15137634 | 13.97717381 | 7.174202538 | 8.34454664 | -1.170344098 | 1.170344098 | 2.25 | 2.41 |
|  |  | 21.13165817 | 14.13511276 | 6.99654541 | 8.34454664 | -1.348001226 | 1.348001226 | 2.55 |  |
|  |  | 21.14768181 | 14.09090328 | 7.056778526 | 8.34454664 | -1.28776811 | 1.28776811 | 2.44 |  |
|  | 5 | 21.58750916 | 14.15561962 | 7.431889534 | 8.34454664 | -0.912657102 | 0.912657102 | 1.88 | 2.01 |
|  |  | 21.5165905 | 14.20073509 | 7.315855408 | 8.34454664 | -1.028691228 | 1.028691228 | 2.04 |  |
|  |  | 21.47415009 | 14.21011925 | 7.264030838 | 8.34454664 | -1.080515798 | 1.080515798 | 2.11 |  |
|  | 6 | 21.97246208 | 14.23030663 | 7.742155457 | 8.34454664 | -0.602391179 | 0.602391179 | 1.52 | 1.46 |
|  |  | 21.98000374 | 14.13459301 | 7.845410728 | 8.34454664 | -0.499135907 | 0.499135907 | 1.41 |  |
|  |  | 22.00251808 | 14.17999363 | 7.822524452 | 8.34454664 | -0.522022184 | 0.522022184 | 1.44 |  |
|  | 7 | 22.04055443 | 14.19079304 | 7.849761391 | 8.34454664 | -0.494785245 | 0.494785245 | 1.41 | 1.41 |
|  |  | 21.96371918 | 14.06950092 | 7.894218254 | 8.34454664 | -0.450328382 | 0.450328382 | 1.37 |  |
|  |  | 21.98248138 | 14.17508602 | 7.807395363 | 8.34454664 | -0.537151273 | 0.537151273 | 1.45 |  |
|  | 8 | 23.00099564 | 13.28041649 | 9.720579147 | 8.34454664 | 1.376032511 | -1.376032511 | 0.39 | 0.28 |
|  |  | 23.55683517 | 13.28260231 | 10.27423286 | 8.34454664 | 1.929686228 | -1.929686228 | 0.26 |  |
|  |  | 23.96620941 | 13.24913788 | 10.71707153 | 8.34454664 | 2.372524897 | -2.372524897 | 0.19 |  |
|  |  |  |  |  |  |  |  |  |  |
| P38 | 1 | 21.47790909 | 12.93815231 | 8.539756775 | 8.61241595 | -0.072659175 | 0.072659175 | 1.05 | 1.00 |
|  |  | 21.56223679 | 12.95792007 | 8.604316711 | 8.61241595 | -0.008099238 | 0.008099238 | 1.01 |  |
|  |  | 21.56461716 | 12.87144279 | 8.693174362 | 8.61241595 | 0.080758413 | -0.080758413 | 0.95 |  |
|  | 2 | 21.4427948 | 14.09781075 | 7.344984055 | 8.61241595 | -1.267431895 | 1.267431895 | 2.41 | 2.31 |
|  |  | 21.55425453 | 14.00054359 | 7.553710938 | 8.61241595 | -1.058705012 | 1.058705012 | 2.08 |  |
|  |  | 21.52225494 | 14.19437695 | 7.327877998 | 8.61241595 | -1.284537951 | 1.284537951 | 2.44 |  |
|  | 3 | 21.6571434 | 13.98803425 | 7.669109154 | 8.61241595 | -0.943306796 | 0.943306796 | 1.92 | 1.95 |
|  |  | 21.76367264 | 14.16033268 | 7.603339958 | 8.61241595 | -1.009075991 | 1.009075991 | 2.01 |  |
|  |  | 21.83022575 | 14.15348434 | 7.676741409 | 8.61241595 | -0.93567454 | 0.93567454 | 1.91 |  |
|  | 4 | 21.56630821 | 13.97717381 | 7.589134407 | 8.61241595 | -1.023281542 | 1.023281542 | 2.03 | 2.23 |
|  |  | 21.48580132 | 14.13511276 | 7.350688553 | 8.61241595 | -1.261727397 | 1.261727397 | 2.40 |  |
|  |  | 21.52943993 | 14.09090328 | 7.438536644 | 8.61241595 | -1.173879306 | 1.173879306 | 2.26 |  |
|  | 5 | 21.84906235 | 14.15561962 | 7.693442726 | 8.61241595 | -0.918973223 | 0.918973223 | 1.89 | 1.87 |
|  |  | 21.99715767 | 14.20073509 | 7.796422577 | 8.61241595 | -0.815993373 | 0.815993373 | 1.76 |  |
|  |  | 21.85797577 | 14.21011925 | 7.647856522 | 8.61241595 | -0.964559428 | 0.964559428 | 1.95 |  |
|  | 6 | 22.16319008 | 14.23030663 | 7.932883453 | 8.61241595 | -0.679532496 | 0.679532496 | 1.60 | 1.67 |
|  |  | 21.99903831 | 14.13459301 | 7.864445305 | 8.61241595 | -0.747970645 | 0.747970645 | 1.68 |  |
|  |  | 22.00679588 | 14.17999363 | 7.826802254 | 8.61241595 | -0.785613696 | 0.785613696 | 1.72 |  |
|  | 7 | 22.02450333 | 14.19079304 | 7.833710289 | 8.61241595 | -0.778705661 | 0.778705661 | 1.72 | 1.62 |
|  |  | 22.06564865 | 14.06950092 | 7.996147728 | 8.61241595 | -0.616268222 | 0.616268222 | 1.53 |  |
|  |  | 22.11258469 | 14.17508602 | 7.937498665 | 8.61241595 | -0.674917285 | 0.674917285 | 1.60 |  |
|  | 8 | 22.62364693 | 13.28041649 | 9.343230438 | 8.61241595 | 0.730814489 | -0.730814489 | 0.60 | 0.60 |
|  |  | 22.69386597 | 13.28260231 | 9.411263657 | 8.61241595 | 0.798847707 | -0.798847707 | 0.57 |  |
|  |  | 22.54910851 | 13.24913788 | 9.299970627 | 8.61241595 | 0.687554677 | -0.687554677 | 0.62 |  |
|  |  |  |  |  |  |  |  |  |  |
| NRF2 | 1 | 20.62581902 | 12.93815231 | 7.687666702 | 7.81059227 | -0.122925568 | 0.122925568 | 1.09 | 1.00 |
|  |  | 20.76010895 | 12.95792007 | 7.802188873 | 7.81059227 | -0.008403397 | 0.008403397 | 1.01 |  |
|  |  | 20.81336403 | 12.87144279 | 7.941921234 | 7.81059227 | 0.131328964 | -0.131328964 | 0.91 |  |
|  | 2 | 25.35189095 | 14.09781075 | 11.2540802 | 7.81059227 | 3.44348793 | -3.44348793 | 0.09 | 0.09 |
|  |  | 25.55463943 | 14.00054359 | 11.55409584 | 7.81059227 | 3.743503571 | -3.743503571 | 0.07 |  |
|  |  | 25.40283966 | 14.19437695 | 11.20846272 | 7.81059227 | 3.397870445 | -3.397870445 | 0.09 |  |
|  | 3 | 22.81953087 | 13.98803425 | 8.83149662 | 7.81059227 | 1.02090435 | -1.02090435 | 0.49 | 0.33 |
|  |  | 22.80542908 | 14.16033268 | 8.645096397 | 7.81059227 | 0.834504128 | -0.834504128 | 0.00 |  |
|  |  | 22.95481834 | 14.15348434 | 8.801334 | 7.81059227 | 0.99074173 | -0.99074173 | 0.50 |  |
|  | 4 | 24.16926155 | 13.97717381 | 10.19208775 | 7.81059227 | 2.381495476 | -2.381495476 | 0.19 | 0.20 |
|  |  | 24.17844048 | 14.13511276 | 10.04332771 | 7.81059227 | 2.232735443 | -2.232735443 | 0.21 |  |
|  |  | 24.23648529 | 14.09090328 | 10.14558201 | 7.81059227 | 2.334989738 | -2.334989738 | 0.20 |  |
|  | 5 | 23.17907486 | 14.15561962 | 9.023455238 | 7.81059227 | 1.212862968 | -1.212862968 | 0.43 | 0.46 |
|  |  | 23.04142456 | 14.20073509 | 8.840689468 | 7.81059227 | 1.030097198 | -1.030097198 | 0.49 |  |
|  |  | 23.16072578 | 14.21011925 | 8.950606537 | 7.81059227 | 1.140014267 | -1.140014267 | 0.45 |  |
|  | 6 | 22.31649628 | 14.23030663 | 8.086189651 | 7.81059227 | 0.275597382 | -0.275597382 | 0.83 | 0.76 |
|  |  | 22.5030838 | 14.13459301 | 8.368490791 | 7.81059227 | 0.557898521 | -0.557898521 | 0.68 |  |
|  |  | 22.35329514 | 14.17999363 | 8.173301506 | 7.81059227 | 0.362709236 | -0.362709236 | 0.78 |  |
|  | 7 | 22.67611237 | 14.19079304 | 8.485319328 | 7.81059227 | 0.674727058 | -0.674727058 | 0.63 | 0.75 |
|  |  | 22.64246292 | 14.06950092 | 8.572961998 | 7.81059227 | 0.762369728 | -0.762369728 | 1.00 |  |
|  |  | 22.63486977 | 14.17508602 | 8.459783745 | 7.81059227 | 0.649191475 | -0.649191475 | 0.64 |  |
|  | 8 | 20.39718475 | 13.28041649 | 7.116768265 | 7.81059227 | -0.693824005 | 0.693824005 | 1.62 | 1.67 |
|  |  | 20.43454552 | 13.28260231 | 7.151943207 | 7.81059227 | -0.658649063 | 0.658649063 | 1.58 |  |
|  |  | 20.19266319 | 13.24913788 | 6.943525314 | 7.81059227 | -0.867066956 | 0.867066956 | 1.82 |  |

S28

|  |  | **目标基因** | **(内参)b-atcin** | **△CT** | **标准参比** | **△△CT** | **—△△CT** | 2—△△CT | 平均值 |
| --- | --- | --- | --- | --- | --- | --- | --- | --- | --- |
| NFKB | 1 | 25.95373 | 12.93815 | 13.01558 | 12.56963 | 0.445946 | -0.44595 | 0.73 | 1.02 |
|  |  | 25.21609 | 12.95792 | 12.25817 | 12.56963 | -0.31146 | 0.311456 | 1.24 |  |
|  |  | 25.30658 | 12.87144 | 12.43514 | 12.56963 | -0.13449 | 0.13449 | 1.10 |  |
|  | 2 | 25.13543 | 14.09781 | 11.03762 | 12.56963 | -1.53201 | 1.532008 | 2.89 | 3.15 |
|  |  | 25.02239 | 14.00054 | 11.02185 | 12.56963 | -1.54778 | 1.547782 | 2.92 |  |
|  |  | 24.90138 | 14.19438 | 10.70701 | 12.56963 | -1.86262 | 1.862624 | 3.64 |  |
|  | 3 | 25.47401 | 14.15562 | 11.31839 | 12.56963 | -1.25124 | 1.251243 | 2.38 | 2.41 |
|  |  | 25.52551 | 14.20074 | 11.32477 | 12.56963 | -1.24486 | 1.244859 | 2.37 |  |
|  |  | 25.47517 | 14.21012 | 11.26505 | 12.56963 | -1.30458 | 1.30458 | 2.47 |  |
|  | 4 | 25.85852 | 14.23031 | 11.62821 | 12.56963 | -0.94142 | 0.941417 | 1.92 | 1.91 |
|  |  | 25.83578 | 14.13459 | 11.70119 | 12.56963 | -0.86844 | 0.868441 | 1.83 |  |
|  |  | 25.76151 | 14.17999 | 11.58152 | 12.56963 | -0.98811 | 0.988114 | 1.98 |  |
|  |  |  |  |  |  |  |  |  |  |
| C0X | 1 | 21.39419 | 12.93815 | 8.456034 | 8.344547 | 0.111487 | -0.11149 | 0.93 | 1.00 |
|  |  | 21.26866 | 12.95792 | 8.310738 | 8.344547 | -0.03381 | 0.033809 | 1.02 |  |
|  |  | 21.13831 | 12.87144 | 8.266869 | 8.344547 | -0.07768 | 0.077678 | 1.06 |  |
|  | 2 | 21.09426 | 14.09781 | 6.996452 | 8.344547 | -1.34809 | 1.348094 | 2.55 | 2.50 |
|  |  | 21.17316 | 14.00054 | 7.172617 | 8.344547 | -1.17193 | 1.17193 | 2.25 |  |
|  |  | 21.09867 | 14.19438 | 6.904292 | 8.344547 | -1.44025 | 1.440255 | 2.71 |  |
|  | 3 | 21.58751 | 14.15562 | 7.43189 | 8.344547 | -0.91266 | 0.912657 | 1.88 | 2.01 |
|  |  | 21.51659 | 14.20074 | 7.315855 | 8.344547 | -1.02869 | 1.028691 | 2.04 |  |
|  |  | 21.47415 | 14.21012 | 7.264031 | 8.344547 | -1.08052 | 1.080516 | 2.11 |  |
|  | 4 | 21.97246 | 14.23031 | 7.742155 | 8.344547 | -0.60239 | 0.602391 | 1.52 | 1.46 |
|  |  | 21.98 | 14.13459 | 7.845411 | 8.344547 | -0.49914 | 0.499136 | 1.41 |  |
|  |  | 22.00252 | 14.17999 | 7.822524 | 8.344547 | -0.52202 | 0.522022 | 1.44 |  |
|  |  |  |  |  |  |  |  |  |  |
| P38 | 1 | 21.47791 | 12.93815 | 8.539757 | 8.612416 | -0.07266 | 0.072659 | 1.05 | 1.00 |
|  |  | 21.56224 | 12.95792 | 8.604317 | 8.612416 | -0.0081 | 0.008099 | 1.01 |  |
|  |  | 21.56462 | 12.87144 | 8.693174 | 8.612416 | 0.080758 | -0.08076 | 0.95 |  |
|  | 2 | 21.44279 | 14.09781 | 7.344984 | 8.612416 | -1.26743 | 1.267432 | 2.41 | 2.31 |
|  |  | 21.55425 | 14.00054 | 7.553711 | 8.612416 | -1.05871 | 1.058705 | 2.08 |  |
|  |  | 21.52225 | 14.19438 | 7.327878 | 8.612416 | -1.28454 | 1.284538 | 2.44 |  |
|  | 3 | 21.84906 | 14.15562 | 7.693443 | 8.612416 | -0.91897 | 0.918973 | 1.89 | 1.87 |
|  |  | 21.99716 | 14.20074 | 7.796423 | 8.612416 | -0.81599 | 0.815993 | 1.76 |  |
|  |  | 21.85798 | 14.21012 | 7.647857 | 8.612416 | -0.96456 | 0.964559 | 1.95 |  |
|  | 4 | 22.16319 | 14.23031 | 7.932883 | 8.612416 | -0.67953 | 0.679532 | 1.60 | 1.67 |
|  |  | 21.99904 | 14.13459 | 7.864445 | 8.612416 | -0.74797 | 0.747971 | 1.68 |  |
|  |  | 22.0068 | 14.17999 | 7.826802 | 8.612416 | -0.78561 | 0.785614 | 1.72 |  |
|  |  |  |  |  |  |  |  |  |  |
| NRF2 | 1 | 20.62582 | 12.93815 | 7.687667 | 7.810592 | -0.12293 | 0.122926 | 1.09 | 1.00 |
|  |  | 20.76011 | 12.95792 | 7.802189 | 7.810592 | -0.0084 | 0.008403 | 1.01 |  |
|  |  | 20.81336 | 12.87144 | 7.941921 | 7.810592 | 0.131329 | -0.13133 | 0.91 |  |
|  | 2 | 25.35189 | 14.09781 | 11.25408 | 7.810592 | 3.443488 | -3.44349 | 0.09 | 0.09 |
|  |  | 25.55464 | 14.00054 | 11.5541 | 7.810592 | 3.743504 | -3.7435 | 0.07 |  |
|  |  | 25.40284 | 14.19438 | 11.20846 | 7.810592 | 3.39787 | -3.39787 | 0.09 |  |
|  | 3 | 23.17907 | 14.15562 | 9.023455 | 7.810592 | 1.212863 | -1.21286 | 0.43 | 0.46 |
|  |  | 23.04142 | 14.20074 | 8.840689 | 7.810592 | 1.030097 | -1.0301 | 0.49 |  |
|  |  | 23.16073 | 14.21012 | 8.950607 | 7.810592 | 1.140014 | -1.14001 | 0.45 |  |
|  | 4 | 22.3165 | 14.23031 | 8.08619 | 7.810592 | 0.275597 | -0.2756 | 0.83 | 0.76 |
|  |  | 22.50308 | 14.13459 | 8.368491 | 7.810592 | 0.557899 | -0.5579 | 0.68 |  |
|  |  | 22.3533 | 14.17999 | 8.173302 | 7.810592 | 0.362709 | -0.36271 | 0.78 |  |

S29

ACTIN
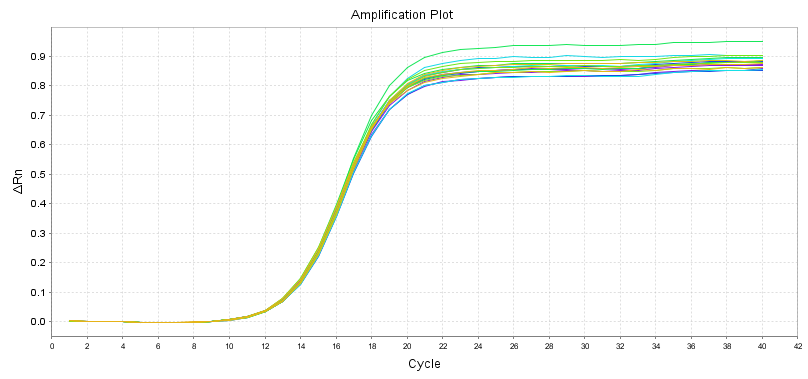


COX-2
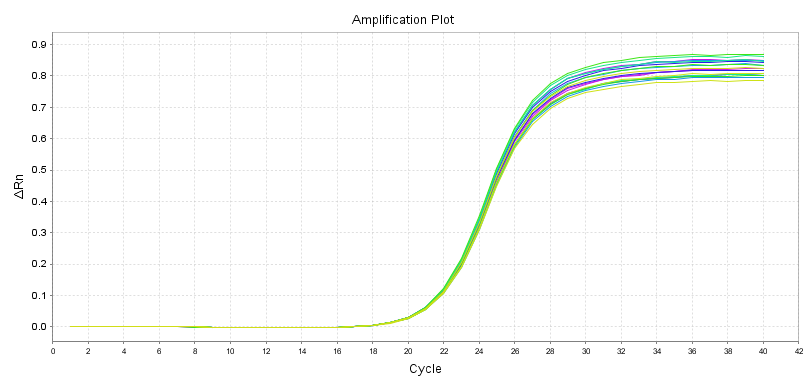


NFkB
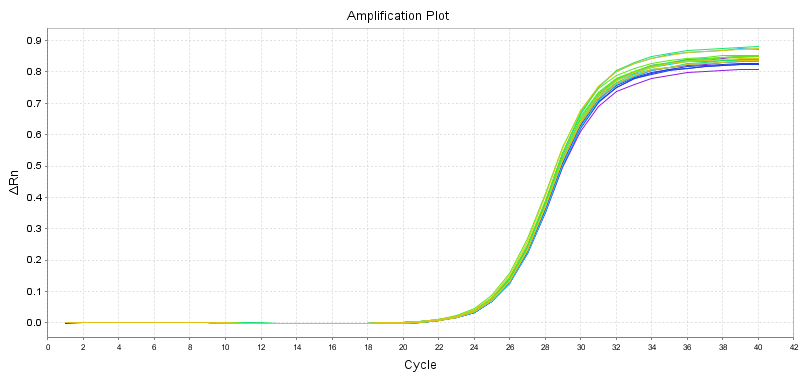


Nrf2
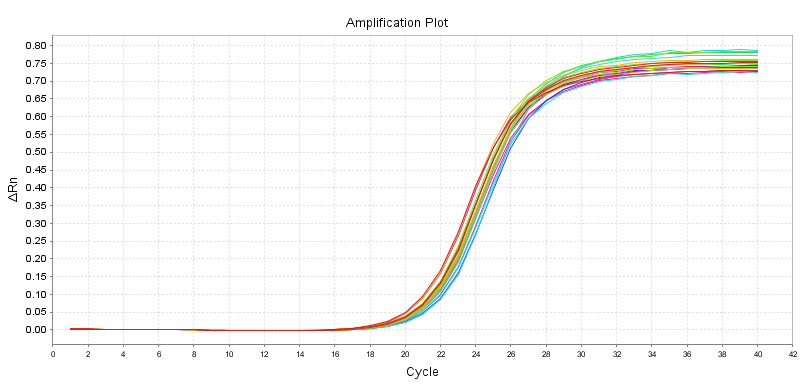


P38
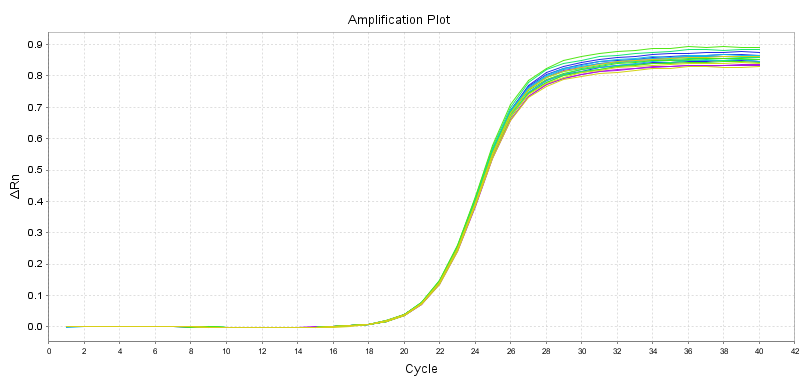


ACTIN
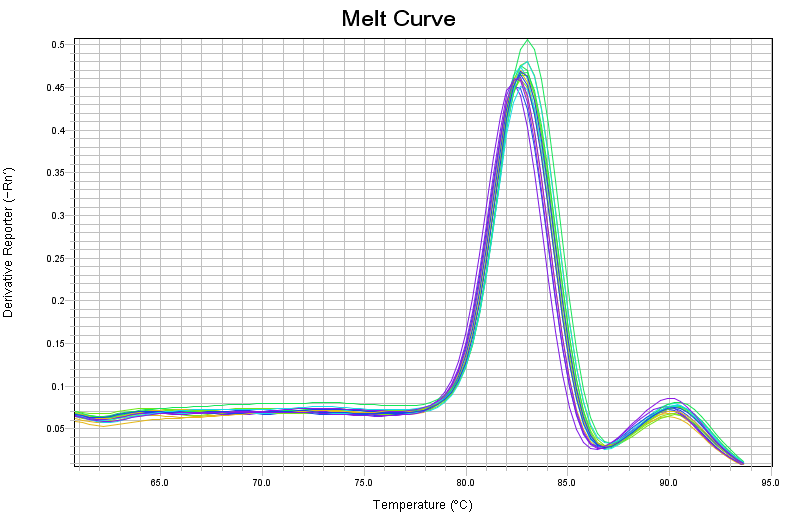


COX-2
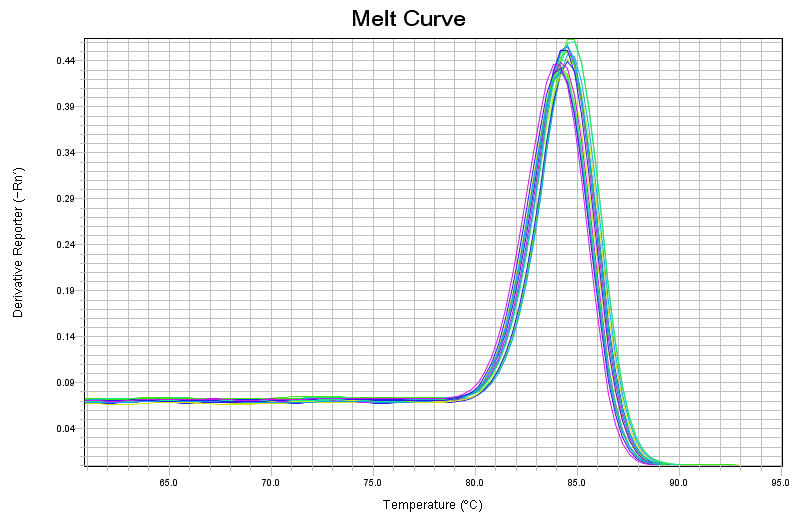


NFkB
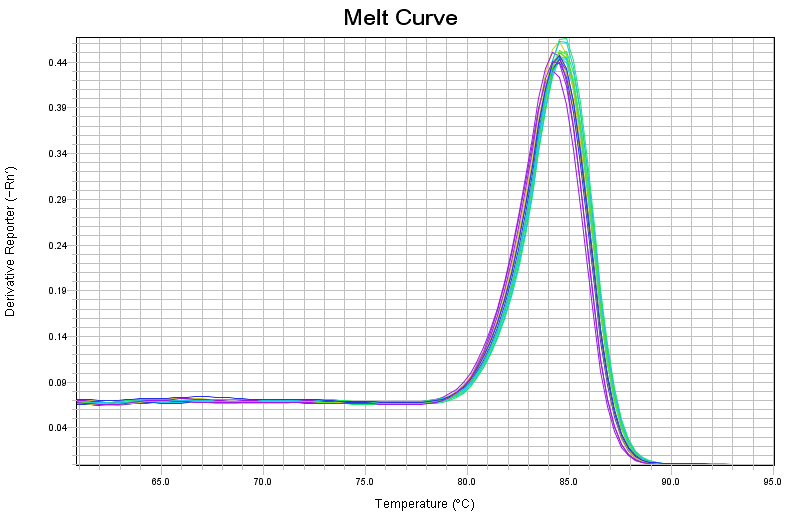


Nrf2


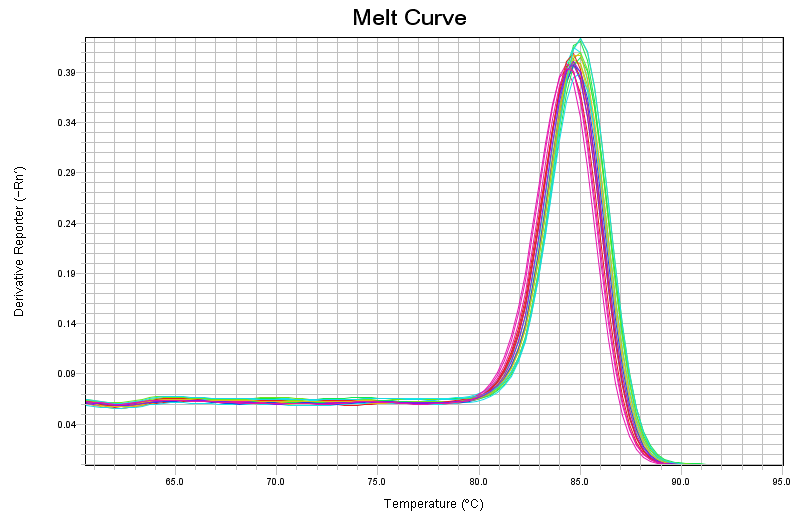


P38
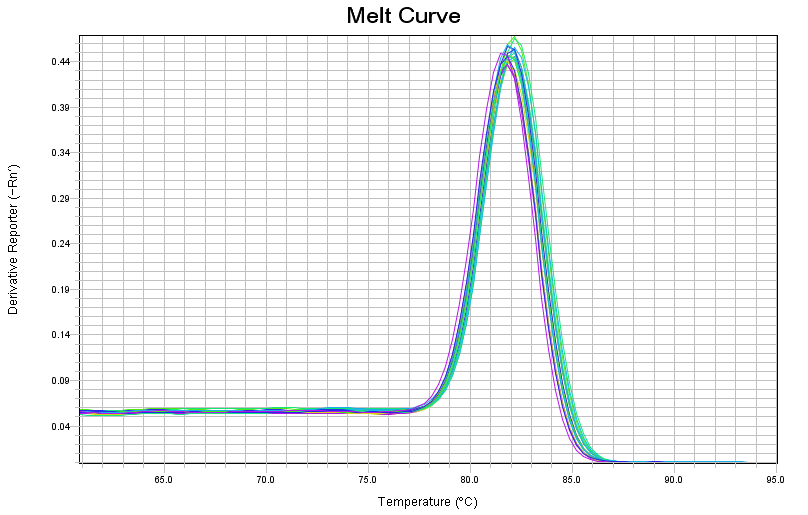


| Well | Sample Name | Target Name | Task | Reporter | Quencher | Cт | Cт Mean | Cт SD | Automatic Ct Threshold | Ct Threshold | Automatic Baseline | Baseline Start | Baseline End | Efficiency | Tm1 | Tm2 | Tm3 | Comments |
| --- | --- | --- | --- | --- | --- | --- | --- | --- | --- | --- | --- | --- | --- | --- | --- | --- | --- | --- |
| A1 | Sample 1 | Target 1 | UNKNOWN | SYBR | None | 20.62582 | 22.71686 | 1.59476 | TRUE | 0.117168 | TRUE | 3 | 17 | 1 | 84.51088 |  |  |  |
| A2 | Sample 1 | Target 1 | UNKNOWN | SYBR | None | 20.76011 | 22.71686 | 1.59476 | TRUE | 0.117168 | TRUE | 3 | 17 | 1 | 84.51088 |  |  |  |
| A3 | Sample 1 | Target 1 | UNKNOWN | SYBR | None | 20.81336 | 22.71686 | 1.59476 | TRUE | 0.117168 | TRUE | 3 | 18 | 1 | 84.68291 |  |  |  |
| B1 | Sample 1 | Target 1 | UNKNOWN | SYBR | None | 25.35189 | 22.71686 | 1.59476 | TRUE | 0.117168 | TRUE | 3 | 18 | 1 | 84.68291 |  |  |  |
| B2 | Sample 1 | Target 1 | UNKNOWN | SYBR | None | 25.55464 | 22.71686 | 1.59476 | TRUE | 0.117168 | TRUE | 3 | 18 | 1 | 84.85493 |  |  |  |
| B3 | Sample 1 | Target 1 | UNKNOWN | SYBR | None | 25.40284 | 22.71686 | 1.59476 | TRUE | 0.117168 | TRUE | 3 | 17 | 1 | 84.85493 |  |  |  |
| C1 | Sample 1 | Target 1 | UNKNOWN | SYBR | None | 22.81953 | 22.71686 | 1.59476 | TRUE | 0.117168 | TRUE | 3 | 18 | 1 | 84.85493 |  |  |  |
| C2 | Sample 1 | Target 1 | UNKNOWN | SYBR | None | 22.80543 | 22.71686 | 1.59476 | TRUE | 0.117168 | TRUE | 3 | 17 | 1 | 85.02695 |  |  |  |
| C3 | Sample 1 | Target 1 | UNKNOWN | SYBR | None | 22.95482 | 22.71686 | 1.59476 | TRUE | 0.117168 | TRUE | 3 | 18 | 1 | 85.02695 |  |  |  |
| D1 | Sample 1 | Target 1 | UNKNOWN | SYBR | None | 24.16926 | 22.71686 | 1.59476 | TRUE | 0.117168 | TRUE | 3 | 18 | 1 | 84.85493 |  |  |  |
| D2 | Sample 1 | Target 1 | UNKNOWN | SYBR | None | 24.17844 | 22.71686 | 1.59476 | TRUE | 0.117168 | TRUE | 3 | 18 | 1 | 85.02695 |  |  |  |
| D3 | Sample 1 | Target 1 | UNKNOWN | SYBR | None | 24.23649 | 22.71686 | 1.59476 | TRUE | 0.117168 | TRUE | 3 | 32 | 1 | 84.85493 |  |  |  |
| E1 | Sample 1 | Target 1 | UNKNOWN | SYBR | None | 23.17907 | 22.71686 | 1.59476 | TRUE | 0.117168 | TRUE | 3 | 18 | 1 | 84.85493 |  |  |  |
| E2 | Sample 1 | Target 1 | UNKNOWN | SYBR | None | 23.04142 | 22.71686 | 1.59476 | TRUE | 0.117168 | TRUE | 3 | 18 | 1 | 84.85493 |  |  |  |
| E3 | Sample 1 | Target 1 | UNKNOWN | SYBR | None | 23.16073 | 22.71686 | 1.59476 | TRUE | 0.117168 | TRUE | 3 | 18 | 1 | 85.02695 |  |  |  |
| F1 | Sample 1 | Target 1 | UNKNOWN | SYBR | None | 22.3165 | 22.71686 | 1.59476 | TRUE | 0.117168 | TRUE | 3 | 31 | 1 | 84.33887 | 75.5658 |  |  |
| F2 | Sample 1 | Target 1 | UNKNOWN | SYBR | None | 22.50308 | 22.71686 | 1.59476 | TRUE | 0.117168 | TRUE | 3 | 18 | 1 | 84.68291 |  |  |  |
| F3 | Sample 1 | Target 1 | UNKNOWN | SYBR | None | 22.3533 | 22.71686 | 1.59476 | TRUE | 0.117168 | TRUE | 3 | 19 | 1 | 84.85493 |  |  |  |
| G1 | Sample 1 | Target 1 | UNKNOWN | SYBR | None | 22.67611 | 22.71686 | 1.59476 | TRUE | 0.117168 | TRUE | 3 | 22 | 1 | 84.51088 |  |  |  |
| G2 | Sample 1 | Target 1 | UNKNOWN | SYBR | None | 22.64246 | 22.71686 | 1.59476 | TRUE | 0.117168 | TRUE | 3 | 18 | 1 | 84.68291 |  |  |  |
| G3 | Sample 1 | Target 1 | UNKNOWN | SYBR | None | 22.63487 | 22.71686 | 1.59476 | TRUE | 0.117168 | TRUE | 3 | 18 | 1 | 84.68291 |  |  |  |
| H1 | Sample 1 | Target 1 | UNKNOWN | SYBR | None | 20.39718 | 22.71686 | 1.59476 | TRUE | 0.117168 | TRUE | 3 | 18 | 1 | 84.33887 |  |  |  |
| H2 | Sample 1 | Target 1 | UNKNOWN | SYBR | None | 20.43455 | 22.71686 | 1.59476 | TRUE | 0.117168 | TRUE | 3 | 17 | 1 | 84.33887 |  |  |  |
| H3 | Sample 1 | Target 1 | UNKNOWN | SYBR | None | 20.19266 | 22.71686 | 1.59476 | TRUE | 0.117168 | TRUE | 3 | 18 | 1 | 84.51088 |  |  |  |

| Well | Sample Name | Target Name | Task | Reporter | Quencher | Cт | Cт Mean | Cт SD | Automatic Ct Threshold | Ct Threshold | Automatic Baseline | Baseline Start | Baseline End | Efficiency | Tm1 |
| --- | --- | --- | --- | --- | --- | --- | --- | --- | --- | --- | --- | --- | --- | --- | --- |
| A4 | Sample 1 | Target 1 | UNKNOWN | SYBR | None | 25.95373 | 23.06644 | 1.895835 | TRUE | 0.080762 | TRUE | 3 | 25 | 1 | 84.18307 |
| A5 | Sample 1 | Target 1 | UNKNOWN | SYBR | None | 25.21609 | 23.06644 | 1.895835 | TRUE | 0.080762 | TRUE | 3 | 25 | 1 | 84.18307 |
| A6 | Sample 1 | Target 1 | UNKNOWN | SYBR | None | 25.30658 | 23.06644 | 1.895835 | TRUE | 0.080762 | TRUE | 3 | 25 | 1 | 84.18307 |
| A7 | Sample 1 | Target 1 | UNKNOWN | SYBR | None | 25.13543 | 23.06644 | 1.895835 | TRUE | 0.080762 | TRUE | 3 | 22 | 1 | 81.82103 |
| A8 | Sample 1 | Target 1 | UNKNOWN | SYBR | None | 25.02239 | 23.06644 | 1.895835 | TRUE | 0.080762 | TRUE | 3 | 22 | 1 | 81.65231 |
| A9 | Sample 1 | Target 1 | UNKNOWN | SYBR | None | 24.90138 | 23.06644 | 1.895835 | TRUE | 0.080762 | TRUE | 3 | 21 | 1 | 81.65231 |
| A10 | Sample 1 | Target 1 | UNKNOWN | SYBR | None | 25.47965 | 23.06644 | 1.895835 | TRUE | 0.080762 | TRUE | 3 | 18 | 1 | 84.3518 |
| A11 | Sample 1 | Target 1 | UNKNOWN | SYBR | None | 25.28573 | 23.06644 | 1.895835 | TRUE | 0.080762 | TRUE | 3 | 18 | 1 | 84.18307 |
| A12 | Sample 1 | Target 1 | UNKNOWN | SYBR | None | 25.46816 | 23.06644 | 1.895835 | TRUE | 0.080762 | TRUE | 3 | 18 | 1 | 84.01436 |
| B4 | Sample 1 | Target 1 | UNKNOWN | SYBR | None | 25.06819 | 23.06644 | 1.895835 | TRUE | 0.080762 | TRUE | 3 | 22 | 1 | 84.52052 |
| B5 | Sample 1 | Target 1 | UNKNOWN | SYBR | None | 25.08081 | 23.06644 | 1.895835 | TRUE | 0.080762 | TRUE | 3 | 21 | 1 | 84.52052 |
| B6 | Sample 1 | Target 1 | UNKNOWN | SYBR | None | 24.90415 | 23.06644 | 1.895835 | TRUE | 0.080762 | TRUE | 3 | 21 | 1 | 84.52052 |
| B7 | Sample 1 | Target 1 | UNKNOWN | SYBR | None | 25.47401 | 23.06644 | 1.895835 | TRUE | 0.080762 | TRUE | 3 | 17 | 1 | 81.98975 |
| B8 | Sample 1 | Target 1 | UNKNOWN | SYBR | None | 25.52551 | 23.06644 | 1.895835 | TRUE | 0.080762 | TRUE | 3 | 18 | 1 | 81.98975 |
| B9 | Sample 1 | Target 1 | UNKNOWN | SYBR | None | 25.47517 | 23.06644 | 1.895835 | TRUE | 0.080762 | TRUE | 3 | 17 | 1 | 81.82103 |
| B10 | Sample 1 | Target 1 | UNKNOWN | SYBR | None | 25.85852 | 23.06644 | 1.895835 | TRUE | 0.080762 | TRUE | 3 | 18 | 1 | 84.68923 |
| B11 | Sample 1 | Target 1 | UNKNOWN | SYBR | None | 25.83578 | 23.06644 | 1.895835 | TRUE | 0.080762 | TRUE | 3 | 18 | 1 | 84.3518 |
| B12 | Sample 1 | Target 1 | UNKNOWN | SYBR | None | 25.76151 | 23.06644 | 1.895835 | TRUE | 0.080762 | TRUE | 3 | 18 | 1 | 84.18307 |
| C4 | Sample 1 | Target 1 | UNKNOWN | SYBR | None | 25.76245 | 23.06644 | 1.895835 | TRUE | 0.080762 | TRUE | 3 | 21 | 1 | 84.52052 |
| C5 | Sample 1 | Target 1 | UNKNOWN | SYBR | None | 25.83662 | 23.06644 | 1.895835 | TRUE | 0.080762 | TRUE | 3 | 21 | 1 | 84.68923 |
| C6 | Sample 1 | Target 1 | UNKNOWN | SYBR | None | 25.82398 | 23.06644 | 1.895835 | TRUE | 0.080762 | TRUE | 3 | 21 | 1 | 84.68923 |
| C7 | Sample 1 | Target 1 | UNKNOWN | SYBR | None | 26.94579 | 23.06644 | 1.895835 | TRUE | 0.080762 | TRUE | 3 | 17 | 1 | 82.15846 |
| C8 | Sample 1 | Target 1 | UNKNOWN | SYBR | None | 26.44378 | 23.06644 | 1.895835 | TRUE | 0.080762 | TRUE | 3 | 17 | 1 | 81.98975 |
| C9 | Sample 1 | Target 1 | UNKNOWN | SYBR | None | 26.82401 | 23.06644 | 1.895835 | TRUE | 0.080762 | TRUE | 3 | 17 | 1 | 81.98975 |
| C10 | Sample 1 | Target 1 | UNKNOWN | SYBR | None | 21.39419 | 23.06644 | 1.895835 | TRUE | 0.080762 | TRUE | 3 | 18 | 1 | 84.68923 |
| C11 | Sample 1 | Target 1 | UNKNOWN | SYBR | None | 21.26866 | 23.06644 | 1.895835 | TRUE | 0.080762 | TRUE | 3 | 18 | 1 | 84.52052 |
| C12 | Sample 1 | Target 1 | UNKNOWN | SYBR | None | 21.13831 | 23.06644 | 1.895835 | TRUE | 0.080762 | TRUE | 3 | 18 | 1 | 84.18307 |
| D4 | Sample 1 | Target 1 | UNKNOWN | SYBR | None | 21.09426 | 23.06644 | 1.895835 | TRUE | 0.080762 | TRUE | 3 | 22 | 1 | 84.68923 |
| D5 | Sample 1 | Target 1 | UNKNOWN | SYBR | None | 21.17316 | 23.06644 | 1.895835 | TRUE | 0.080762 | TRUE | 3 | 21 | 1 | 84.68923 |
| D6 | Sample 1 | Target 1 | UNKNOWN | SYBR | None | 21.09867 | 23.06644 | 1.895835 | TRUE | 0.080762 | TRUE | 3 | 21 | 1 | 84.68923 |
| D7 | Sample 1 | Target 1 | UNKNOWN | SYBR | None | 21.23416 | 23.06644 | 1.895835 | TRUE | 0.080762 | TRUE | 3 | 17 | 1 | 82.15846 |
| D8 | Sample 1 | Target 1 | UNKNOWN | SYBR | None | 21.32006 | 23.06644 | 1.895835 | TRUE | 0.080762 | TRUE | 3 | 17 | 1 | 82.15846 |
| D9 | Sample 1 | Target 1 | UNKNOWN | SYBR | None | 21.29846 | 23.06644 | 1.895835 | TRUE | 0.080762 | TRUE | 3 | 17 | 1 | 81.98975 |
| D10 | Sample 1 | Target 1 | UNKNOWN | SYBR | None | 21.15138 | 23.06644 | 1.895835 | TRUE | 0.080762 | TRUE | 3 | 18 | 1 | 84.68923 |
| D11 | Sample 1 | Target 1 | UNKNOWN | SYBR | None | 21.13166 | 23.06644 | 1.895835 | TRUE | 0.080762 | TRUE | 3 | 18 | 1 | 84.52052 |
| D12 | Sample 1 | Target 1 | UNKNOWN | SYBR | None | 21.14768 | 23.06644 | 1.895835 | TRUE | 0.080762 | TRUE | 3 | 18 | 1 | 84.18307 |
| E4 | Sample 1 | Target 1 | UNKNOWN | SYBR | None | 21.58751 | 23.06644 | 1.895835 | TRUE | 0.080762 | TRUE | 3 | 22 | 1 | 84.68923 |
| E5 | Sample 1 | Target 1 | UNKNOWN | SYBR | None | 21.51659 | 23.06644 | 1.895835 | TRUE | 0.080762 | TRUE | 3 | 21 | 1 | 84.68923 |
| E6 | Sample 1 | Target 1 | UNKNOWN | SYBR | None | 21.47415 | 23.06644 | 1.895835 | TRUE | 0.080762 | TRUE | 3 | 21 | 1 | 84.68923 |
| E7 | Sample 1 | Target 1 | UNKNOWN | SYBR | None | 21.97246 | 23.06644 | 1.895835 | TRUE | 0.080762 | TRUE | 3 | 17 | 1 | 82.15846 |
| E8 | Sample 1 | Target 1 | UNKNOWN | SYBR | None | 21.98 | 23.06644 | 1.895835 | TRUE | 0.080762 | TRUE | 3 | 17 | 1 | 81.98975 |
| E9 | Sample 1 | Target 1 | UNKNOWN | SYBR | None | 22.00252 | 23.06644 | 1.895835 | TRUE | 0.080762 | TRUE | 3 | 17 | 1 | 81.82103 |
| E10 | Sample 1 | Target 1 | UNKNOWN | SYBR | None | 22.04055 | 23.06644 | 1.895835 | TRUE | 0.080762 | TRUE | 3 | 18 | 1 | 84.68923 |
| E11 | Sample 1 | Target 1 | UNKNOWN | SYBR | None | 21.96372 | 23.06644 | 1.895835 | TRUE | 0.080762 | TRUE | 3 | 18 | 1 | 84.52052 |
| E12 | Sample 1 | Target 1 | UNKNOWN | SYBR | None | 21.98248 | 23.06644 | 1.895835 | TRUE | 0.080762 | TRUE | 3 | 18 | 1 | 84.18307 |
| F4 | Sample 1 | Target 1 | UNKNOWN | SYBR | None | 23.001 | 23.06644 | 1.895835 | TRUE | 0.080762 | TRUE | 3 | 22 | 1 | 84.52052 |
| F5 | Sample 1 | Target 1 | UNKNOWN | SYBR | None | 23.55684 | 23.06644 | 1.895835 | TRUE | 0.080762 | TRUE | 3 | 22 | 1 | 84.52052 |
| F6 | Sample 1 | Target 1 | UNKNOWN | SYBR | None | 23.96621 | 23.06644 | 1.895835 | TRUE | 0.080762 | TRUE | 3 | 22 | 1 | 84.52052 |
| F7 | Sample 1 | Target 1 | UNKNOWN | SYBR | None | 21.47791 | 23.06644 | 1.895835 | TRUE | 0.080762 | TRUE | 3 | 17 | 1 | 81.98975 |
| F8 | Sample 1 | Target 1 | UNKNOWN | SYBR | None | 21.56224 | 23.06644 | 1.895835 | TRUE | 0.080762 | TRUE | 3 | 17 | 1 | 81.98975 |
| F9 | Sample 1 | Target 1 | UNKNOWN | SYBR | None | 21.56462 | 23.06644 | 1.895835 | TRUE | 0.080762 | TRUE | 3 | 17 | 1 | 81.82103 |
| F10 | Sample 1 | Target 1 | UNKNOWN | SYBR | None | 21.44279 | 23.06644 | 1.895835 | TRUE | 0.080762 | TRUE | 3 | 18 | 1 | 84.52052 |
| F11 | Sample 1 | Target 1 | UNKNOWN | SYBR | None | 21.55425 | 23.06644 | 1.895835 | TRUE | 0.080762 | TRUE | 3 | 18 | 1 | 84.3518 |
| F12 | Sample 1 | Target 1 | UNKNOWN | SYBR | None | 21.52225 | 23.06644 | 1.895835 | TRUE | 0.080762 | TRUE | 3 | 18 | 1 | 84.18307 |
| G4 | Sample 1 | Target 1 | UNKNOWN | SYBR | None | 21.65714 | 23.06644 | 1.895835 | TRUE | 0.080762 | TRUE | 3 | 22 | 1 | 84.18307 |
| G5 | Sample 1 | Target 1 | UNKNOWN | SYBR | None | 21.76367 | 23.06644 | 1.895835 | TRUE | 0.080762 | TRUE | 3 | 21 | 1 | 84.3518 |
| G6 | Sample 1 | Target 1 | UNKNOWN | SYBR | None | 21.83023 | 23.06644 | 1.895835 | TRUE | 0.080762 | TRUE | 3 | 21 | 1 | 84.3518 |
| G7 | Sample 1 | Target 1 | UNKNOWN | SYBR | None | 21.56631 | 23.06644 | 1.895835 | TRUE | 0.080762 | TRUE | 3 | 17 | 1 | 81.82103 |
| G8 | Sample 1 | Target 1 | UNKNOWN | SYBR | None | 21.4858 | 23.06644 | 1.895835 | TRUE | 0.080762 | TRUE | 3 | 17 | 1 | 81.82103 |
| G9 | Sample 1 | Target 1 | UNKNOWN | SYBR | None | 21.52944 | 23.06644 | 1.895835 | TRUE | 0.080762 | TRUE | 3 | 17 | 1 | 81.65231 |
| G10 | Sample 1 | Target 1 | UNKNOWN | SYBR | None | 21.84906 | 23.06644 | 1.895835 | TRUE | 0.080762 | TRUE | 3 | 18 | 1 | 84.3518 |
| G11 | Sample 1 | Target 1 | UNKNOWN | SYBR | None | 21.99716 | 23.06644 | 1.895835 | TRUE | 0.080762 | TRUE | 3 | 18 | 1 | 84.18307 |
| G12 | Sample 1 | Target 1 | UNKNOWN | SYBR | None | 21.85798 | 23.06644 | 1.895835 | TRUE | 0.080762 | TRUE | 3 | 18 | 1 | 84.01436 |
| H4 | Sample 1 | Target 1 | UNKNOWN | SYBR | None | 22.16319 | 23.06644 | 1.895835 | TRUE | 0.080762 | TRUE | 3 | 28 | 1 | 83.84564 |
| H5 | Sample 1 | Target 1 | UNKNOWN | SYBR | None | 21.99904 | 23.06644 | 1.895835 | TRUE | 0.080762 | TRUE | 3 | 29 | 1 | 84.01436 |
| H6 | Sample 1 | Target 1 | UNKNOWN | SYBR | None | 22.0068 | 23.06644 | 1.895835 | TRUE | 0.080762 | TRUE | 3 | 28 | 1 | 84.01436 |
| H7 | Sample 1 | Target 1 | UNKNOWN | SYBR | None | 22.0245 | 23.06644 | 1.895835 | TRUE | 0.080762 | TRUE | 3 | 25 | 1 | 81.65231 |
| H8 | Sample 1 | Target 1 | UNKNOWN | SYBR | None | 22.06565 | 23.06644 | 1.895835 | TRUE | 0.080762 | TRUE | 3 | 25 | 1 | 81.48359 |
| H9 | Sample 1 | Target 1 | UNKNOWN | SYBR | None | 22.11258 | 23.06644 | 1.895835 | TRUE | 0.080762 | TRUE | 3 | 25 | 1 | 81.31487 |
| H10 | Sample 1 | Target 1 | UNKNOWN | SYBR | None | 22.62365 | 23.06644 | 1.895835 | TRUE | 0.080762 | TRUE | 3 | 18 | 1 | 84.18307 |
| H11 | Sample 1 | Target 1 | UNKNOWN | SYBR | None | 22.69387 | 23.06644 | 1.895835 | TRUE | 0.080762 | TRUE | 3 | 18 | 1 | 84.01436 |
| H12 | Sample 1 | Target 1 | UNKNOWN | SYBR | None | 22.54911 | 23.06644 | 1.895835 | TRUE | 0.080762 | TRUE | 3 | 18 | 1 | 83.84564 |

| Well | Sample Name | Target Name | Task | Reporter | Quencher | #NAME? | Cт Mean | Cт SD | Automatic Ct Threshold | Ct Threshold | Automatic Baseline | Baseline Start | Baseline End | Efficiency | Tm1 | Tm2 |
| --- | --- | --- | --- | --- | --- | --- | --- | --- | --- | --- | --- | --- | --- | --- | --- | --- |
| A1 | Sample 1 | NRF2 | UNKNOWN | SYBR | None | 12.93815 | 13.87184 | 0.470756 | TRUE | 0.150376 | TRUE | 3 | 8 | 1 | 82.34404 | 90.03835 |
| A2 | Sample 1 | NRF2 | UNKNOWN | SYBR | None | 12.95792 | 13.87184 | 0.470756 | TRUE | 0.150376 | TRUE | 3 | 8 | 1 | 82.34404 | 90.03835 |
| A3 | Sample 1 | NRF2 | UNKNOWN | SYBR | None | 12.87144 | 13.87184 | 0.470756 | TRUE | 0.150376 | TRUE | 3 | 8 | 1 | 82.51503 | 90.20933 |
| B1 | Sample 1 | NRF2 | UNKNOWN | SYBR | None | 14.09781 | 13.87184 | 0.470756 | TRUE | 0.150376 | TRUE | 3 | 9 | 1 | 82.51503 |  |
| B2 | Sample 1 | NRF2 | UNKNOWN | SYBR | None | 14.00054 | 13.87184 | 0.470756 | TRUE | 0.150376 | TRUE | 3 | 9 | 1 | 82.68601 |  |
| B3 | Sample 1 | NRF2 | UNKNOWN | SYBR | None | 14.19438 | 13.87184 | 0.470756 | TRUE | 0.150376 | TRUE | 3 | 9 | 1 | 82.68601 | 90.20933 |
| C1 | Sample 1 | NRF2 | UNKNOWN | SYBR | None | 13.98803 | 13.87184 | 0.470756 | TRUE | 0.150376 | TRUE | 3 | 9 | 1 | 82.68601 |  |
| C2 | Sample 1 | NRF2 | UNKNOWN | SYBR | None | 14.16033 | 13.87184 | 0.470756 | TRUE | 0.150376 | TRUE | 3 | 9 | 1 | 82.85699 |  |
| C3 | Sample 1 | NRF2 | UNKNOWN | SYBR | None | 14.15348 | 13.87184 | 0.470756 | TRUE | 0.150376 | TRUE | 3 | 9 | 1 | 82.85699 | 90.38031 |
| D1 | Sample 1 | NRF2 | UNKNOWN | SYBR | None | 13.97717 | 13.87184 | 0.470756 | TRUE | 0.150376 | TRUE | 3 | 9 | 1 | 82.85699 | 90.20933 |
| D2 | Sample 1 | NRF2 | UNKNOWN | SYBR | None | 14.13511 | 13.87184 | 0.470756 | TRUE | 0.150376 | TRUE | 3 | 9 | 1 | 82.85699 | 90.38031 |
| D3 | Sample 1 | NRF2 | UNKNOWN | SYBR | None | 14.0909 | 13.87184 | 0.470756 | TRUE | 0.150376 | TRUE | 3 | 9 | 1 | 83.02798 | 90.38031 |
| E1 | Sample 1 | NRF2 | UNKNOWN | SYBR | None | 14.15562 | 13.87184 | 0.470756 | TRUE | 0.150376 | TRUE | 3 | 9 | 1 | 82.68601 | 90.20933 |
| E2 | Sample 1 | NRF2 | UNKNOWN | SYBR | None | 14.20074 | 13.87184 | 0.470756 | TRUE | 0.150376 | TRUE | 3 | 9 | 1 | 82.85699 | 90.20933 |
| E3 | Sample 1 | NRF2 | UNKNOWN | SYBR | None | 14.21012 | 13.87184 | 0.470756 | TRUE | 0.150376 | TRUE | 3 | 9 | 1 | 82.85699 | 90.20933 |
| F1 | Sample 1 | NRF2 | UNKNOWN | SYBR | None | 14.23031 | 13.87184 | 0.470756 | TRUE | 0.150376 | TRUE | 3 | 9 | 1 | 82.51503 | 89.86736 |
| F2 | Sample 1 | NRF2 | UNKNOWN | SYBR | None | 14.13459 | 13.87184 | 0.470756 | TRUE | 0.150376 | TRUE | 3 | 9 | 1 | 82.68601 | 90.03835 |
| F3 | Sample 1 | NRF2 | UNKNOWN | SYBR | None | 14.17999 | 13.87184 | 0.470756 | TRUE | 0.150376 | TRUE | 3 | 9 | 1 | 82.68601 | 90.20933 |
| G1 | Sample 1 | NRF2 | UNKNOWN | SYBR | None | 14.19079 | 13.87184 | 0.470756 | TRUE | 0.150376 | TRUE | 3 | 9 | 1 | 82.34404 | 89.69637 |
| G2 | Sample 1 | NRF2 | UNKNOWN | SYBR | None | 14.0695 | 13.87184 | 0.470756 | TRUE | 0.150376 | TRUE | 3 | 9 | 1 | 82.51503 | 89.86736 |
| G3 | Sample 1 | NRF2 | UNKNOWN | SYBR | None | 14.17509 | 13.87184 | 0.470756 | TRUE | 0.150376 | TRUE | 3 | 9 | 1 | 82.51503 | 90.03835 |
| H1 | Sample 1 | NRF2 | UNKNOWN | SYBR | None | 13.28042 | 13.87184 | 0.470756 | TRUE | 0.150376 | TRUE | 3 | 8 | 1 | 82.17306 | 89.52539 |
| H2 | Sample 1 | NRF2 | UNKNOWN | SYBR | None | 13.2826 | 13.87184 | 0.470756 | TRUE | 0.150376 | TRUE | 3 | 8 | 1 | 82.17306 | 89.69637 |
| H3 | Sample 1 | NRF2 | UNKNOWN | SYBR | None | 13.24914 | 13.87184 | 0.470756 | TRUE | 0.150376 | TRUE | 3 | 8 | 1 | 82.34404 | 89.86736 |
